# Family‐Related Factors in Children and Adolescents With Chronic Pain: A Systematic Review

**DOI:** 10.1002/ejp.70038

**Published:** 2025-05-23

**Authors:** Lorena Martí, Helena Garriga‐Cazorla, Josep Roman‐Juan, Jordi Miró

**Affiliations:** ^1^ Unit for the Study and Treatment of Pain – ALGOS Universitat Rovira i Virgili Catalonia Spain; ^2^ Department of Psychology Research Center for Behaviour Assessment (CRAMC) Catalonia Spain; ^3^ Institut d'Investigació Sanitària Pere Virgili, Universitat Rovira i Virgili Catalonia Spain

**Keywords:** adolescents, children, chronic pain, family‐related factors, systematic review

## Abstract

**Background:**

The aim of this systematic review is to synthesise the findings about the role of family‐related factors in chronic pain in children and adolescents.

**Methods:**

We conducted a search of the following electronic databases: PubMed/MedLine, CINHAL, PsychINFO, PubPsych, Scopus and Web of Science from inception to July 2024. We only included studies involving children and adolescents (up to 19 years old) with chronic pain and studies that involved the parents or families of these children.

**Results:**

A total of 24,049 articles were retrieved, of which 20,921 were screened for evaluation and 119 were included in the review.

**Conclusions:**

Most of the studies were cross‐sectional with a moderate or high risk of bias, reporting on the parenting individual‐, dyadic‐, and contextual‐related factors. In the included studies, significant associations emerged between a number of family‐related factors and chronic pain and related disability in 16 of the 119 studies that had been judged to be of good methodological quality.

**Significance:**

The data from these studies showed significant associations between parental individual variables (e.g., parent's mental health), dyadic variables (e.g., parental responses towards their children's pain), and context‐related variables (e.g., family functioning), and some key pain‐related outcomes, including pain chronification, pain intensity, pain frequency, pain extent, pain‐related interference, the ability to cope with chronic pain, and pain‐related disability in children and adolescents. Therefore, these factors may be important targets for the prevention and management of chronic pain in children and adolescents.

## 1 Introduction

Chronic pain has been defined as pain that lasts or recurs for 3 months or longer (Treede et al. 2015). Chronic pain can be primary (i.e., not better accounted for by a specific classified disease, e.g., complex regional pain syndrome) or secondary (linked to an underlying condition, e.g., chronic postsurgical pain). It is a common and significant problem in children and adolescents (Miró et al. 2022) associated with increased risk of psychological problems (Simons et al. 2012) and reduced quality of life (Gold et al. 2009). Although estimates vary, research indicates high and rising prevalence rates (Roy et al. 2021; Roman‐Juan et al. 2023), including complex, high‐impact cases (Miró et al. 2023). For example, in a recent review, Chambers et al. (2024) reported a 21% prevalence.

The biopsychosocial model considers chronic pain as a complex interplay of internal and external personal characteristics (Miró 2010). These include physical factors such as pain intensity (De la Vega et al. 2016; Hechler et al. 2011), pain extension (i.e., the number of body areas with pain; Gobina et al. 2019), or inflammation (Castori and Hakim 2017), as well as psychological factors such as emotions (Tomé‐Pires et al. 2016) or cognitions (Solé et al. 2016), and social factors such as adult reactions to a child's pain behaviours (Huguet and Miró 2008) or family dynamics (Miró et al. 2019).

Even though studies on social factors in chronic paediatric pain lag behind studies on physical and psychological factors, existing evidence highlights their role in adjusting to and coping with chronic pain in paediatric samples (Cordts et al. 2016; Miró et al. 2019). Family‐related factors are gaining attention as key influences on chronic pain in children and adolescents (Cordts et al. 2016; Grasaas et al. 2023; Miró et al. 2007; Palermo et al. 2014). Recent reviews (e.g., Donnelly et al. 2020; Beveridge et al. 2023) have explored the role of family‐related factors in chronic pain in children and adolescents in a limited way; these studies primarily focused on mental health and individual parent cognitive, behavioural, and affective factors. As a result, many family‐related factors have yet to be examined from this perspective. Clinicians could use the findings to decide which of the modifiable factors should be the target of future interventions or—if there are no significant associations—not be considered at all. They could also be used to determine the factors or variables that merit closer examination in future experimental studies, which could, in turn, improve the understanding of how they are contributing to the experience of chronic pain in young individuals. Furthermore, past reviews have not assessed the methodological quality of the included studies, leaving unclear whether their conclusions are based on robust research. Thus, the aims of this systematic review were to (1) synthesise the findings on family‐related factors in paediatric chronic pain focusing on pain intensity, frequency, disability, and adjustment to chronic pain and (2) evaluate the quality of the studies.

## 2 Methods

### 2.1 Search Strategy and Identification of Studies

We searched the electronic databases PubMed/MedLine, CINAHL, PsychINFO, PubPsych, Scopus, and Web of Science to identify relevant studies. The initial search included all records published before June 2023. A subsequent search was then conducted just before this paper was submitted for consideration for publication on 17 July 2024. The search terms used were the following: (“chronic pain” OR “musculoskeletal pain” OR “back pain” OR “abdominal pain” OR “headache” OR “migraine” OR “back pain” OR “arthritis” OR “complex regional pain syndrome” OR “fibromyalgia”) AND (family OR parent* OR socio* OR cultur* OR context* OR econom* OR father OR mother) AND (child* OR adolesc* OR youth* OR young* OR paediatric* OR paediatric*) (asterisks were used as wildcards to search terms with the same word root). Reference lists of relevant retrieved papers were also reviewed to identify any other published works not found in the computerised database searches. Moreover, we also conducted a grey literature search (e.g., documents such as reports, policy papers, working papers, conference proceedings, theses, dissertations, government publications, or technical standards).

### 2.2 Inclusion Criteria

A study was included in the review if (1) it was a cohort study, case–control study, longitudinal study, or cross‐sectional study; (2) it was published in a peer‐reviewed journal; (3) one or more family‐related factors were included in the analyses; (4) participants were children and adolescents (up to 19 years old) with chronic pain or parents/family of children with chronic pain; and (5) it was written in English or Spanish. We excluded studies using single‐item questions; systematic reviews; case reports; qualitative studies; intervention studies; observational studies; practice guidelines; commentaries; articles focusing on the validation of family function measures; and studies of children and adolescents with acute pain or cancer‐related chronic pain, or chronic pain in individuals with intellectual disabilities.

This review was registered in PROSPERO (ref. number: CRD42021240140) and conducted in accordance with the PRISMA recommendations for reporting systematic reviews and meta‐analyses (Page et al. 2021). The articles were evaluated by three researchers (L.M., H.G‐C., and J.R‐J.) to determine whether they met the inclusion criteria, first by screening the abstracts and then by reviewing the full text. If there was any disagreement about eligibility for inclusion, a fourth researcher (J.M.) was brought in, and any disagreements were discussed until consensus was reached.

### 2.3 Data Extraction and Analysis

The following information was collected for each study: author/s, publication date, study objective(s), description of sample, type of pain, study design, family factors included, questionnaires used to assess family‐related factors, results regarding pain outcomes, and the risk of bias.

A narrative analysis of the findings structured around the key family‐related factors (i.e., parental individual factors, dyadic factors, and context‐related factors) and descriptive characteristics of the targeted population is provided. In addition, the family factors related to chronic pain in children and adolescents are summarised in tabular form.

### 2.4 Risk of Bias and Quality Assessment

The pool of articles meeting the eligibility criteria was appraised for risk of bias using two reliable and valid instruments for assessing the quality of studies: the Newcastle‐Ottawa Quality Assessment Scale (NOS) for longitudinal cohort studies and case–control studies (Wells et al. 2003) and the National Institute of Health (NIH) quality assessment tool for observational cohort and cross‐sectional studies (NIH 2014). The assessment covered the representativeness and selection of the cohort or the definition of the case (selection domain), the comparability of cohorts (comparability domain), and the evaluation and follow‐up of outcomes/exposure (exposure domain). The NOS awarded a point for each answer that was marked with an asterisk (*) in accordance with the instructions on the coding manual (see https://www.ohri.ca/programs/clinical_epidemiology/nos_manual.pdf), and the results were converted into the Agency for Healthcare Research and Quality standards of ‘good’, ‘fair’, and ‘poor’ quality. As no explicit conversion guidance exists, conversion thresholds from previous publications were used (Barthold and González 2003; Robinson et al. 2021): that is to say, three or four asterisks in the selection domain, one or two asterisks in the comparability domain, and two or three asterisks in the exposure domain were rated as good quality; two asterisks in the selection domain, one or two asterisks in the comparability domain, and two or three asterisks in the exposure domain were rated as fair quality; and zero or one asterisk in the selection domain or zero asterisks in the comparability domain or zero or one asterisk in the exposure domain were rated as poor quality. Using the NIH observational assessment tool, the methodological criteria were scored as “yes” (1 point) and “no” or “not reported” (0 points). For each study reviewed, the scores were added together to provide an indicator of the methodological quality. So, following procedures used in similar published reviews (Farley et al. 2020; Lyons et al. 2017; Macdonald et al. 2018) and using cutoff points at 50% and 75%, the indicator ranged from 0 to 6 (poor), 7 to 10 (fair), and 11 to 14 (good). Detailed information about the methodological quality of the studies reviewed is provided in Tables 1, 2, 3.

**TABLE 1:** Methodological quality of longitudinal cohort studies—Newcastle Ottawa scale results.

First author, year	Selection				Comparability	Exposure			Quality rating*
	Representativeness of the exposed cohort	Selection of the non‐exposed cohort	Ascertainment of exposed cohort	Demonstration that outcome of interest was not present at start of study	Comparability of cohorts on the basis of the design of analysis	Assessment of outcome	Was follow‐up long enough for outcomes to occur?	Adequacy of follow up of cohorts	
Aasland et al. (1997)	A*	A*	A*	A*	B*	A*	A*	D	Good
Beeckman, Hughes, et al. (2019)	B*	A*	C	A*	A*	C	A*	A*	Good
Carasco and Kröner‐Herwig (2016)	A*	A*	D	A*	A*	C	A*	D	Poor
Chow et al. (2016)	B*	A*	C	A*	B*	C	A*	C	Poor
Crushell et al. (2003)	B*	A*	B*	A*		C	A*	A*	Poor
Dougherty et al. (2021)	B*	A*	C	A*	A*	C	A*	C	Poor
Hammond et al. (2019)	A*	A*	B*	A*	A*	C	A*	A*	Good
Helgeland et al. (2010)	A*	A*	C	A*	A*	C	A*	A*	Fair
Incledon et al. (2016)	A*	A*	C	A*	A*	C	A*	A*	Fair
Larsson and Sund (2007)	A*	A*	C	A*		C	A*	A*	Fair
Law et al. (2020)	B*	A*	C	A*	A*	C	A*	A*	Good
Nelson et al. (2021)	A*	C	C	A*		C	A*	B*	Fair
Petri et al. (2024)	A*	A*	C	A*	A*	C	A*	B*	Good
Timko et al. (1993)	B*	A*	C	A*	A*	C	A*	A*	Good
Toupin April et al. (2012)	B*	A*	C	A*	A*	C	A*	C	Fair
Welkom et al. (2013)	B*	A*	C	A*		C	A*	B*	Poor

* Thresholds for converting the Newcastle–Ottawa scales to AHRQ standards (good, fair and poor): Good quality: three or four stars in selection domain AND one or two stars in comparability domain AND two or three stars in exposure domain; Fair quality: two stars in selection domain AND one or two stars in comparability domain AND two or three stars in exposure domain; Poor quality: zero or one star in selection domain OR zero stars in comparability domain OR zero or one star in exposure domain.

**TABLE 2:** Methodological quality of case–control studies—Newcastle Ottawa scale results.

First author, year	Selection				Comparability	Outcome			Quality rating*
	The case definition is adequate	Representativeness of the cases	Selection of controls	Definition of controls	Comparability of cases and controls on the basis of the design or analysis	Ascertainment of exposure	Same method of ascertainment for cases and controls	Non‐response rate	
Aromaa et al. (1998)	A*	A*	B	B	A*	A*	A*	A*	Fair
Beveridge et al. (2020)	A*	B	A*	B	A*	D	A*	C	Poor
Bizzi et al. (2020)	A*	B	A*	A*	A*	B*	A*	A*	Good
Clementi et al. (2019)	A*	B	B	A*	A*	D	A*	C	Poor
Coenders et al. (2014)	A*	B	B	A*	C	D	A*	C	Poor
Conte et al. (2003)	A*	A*	B	A*	B*	D	A*	A*	Fair
Ertem et al. (2019)	A*	A*	A*	A*	A*	A*	A*	C	Good
Esposito et al. (2013)	A*	A*	A*	A*	A*	D	A*	C	Poor
Evans et al. (2018)	A*	B	A*	A*	A*	D	A*	C	Poor
Heikkala et al. (2023)	A*	A*	B	B	A*	D	A*	B	Poor
Ioannis et al. (2013)	A*	B	A*	A*	B*	D	A*	C	Poor
Juang et al. (2004)	A*	A*	A*	A*	A*	B*	A*	A*	Good
Kandemir et al. (2018)	A*	B	B	A*	A*	D	A*	C	Poor
Kashikar‐Zuck et al. (2008)	A*	B	A*	A*	A*	D	A*	A*	Good
Laurell et al. (2005)	A*	A*	A*	A*	A*	D	A*	A*	Good
Liakopoulou‐Kairis et al. (2002)	A*	B	B	A*	D	C	A*	A*	Poor
Neufeld et al. (2013)	A*	A*	A*	A*	A*	D	A*	C	Poor
Reid et al. (1997)	A*	B	A*	A*	A*	D	A*	C	Poor
Shenoi et al. (2016)	A*	B	C	A*	A*	D	A*	C	Poor
Siniatchkin et al. (2003)	A*	B	A*	A*	D	A*	A*	C	Poor
Wilson et al. (2020)	A*	A*	A*	A*	A*	D	A*	A*	Good
Yadav and Yadav (2013)	A*	B	C	A*	B*	D	A*	C	Poor

* Thresholds for converting the Newcastle–Ottawa scales to AHRQ standards (good, fair and poor): Good quality: three or four stars in selection domain AND one or two stars in comparability domain AND two or three stars in outcome domain. Fair quality: two stars in selection domain AND one or two stars in comparability domain AND two or three stars in outcome domain. Poor quality: zero or one star in selection domain OR zero stars in comparability domain OR zero or one star in outcome domain.

**TABLE 3:** Methodological quality of observational cohort and cross‐sectional studies—The National Institutes of Health quality assessment tool.

First author, year	Study objectives stated	Study population defined	Eligible participation rate at least 50%?	Participant selection and inclusion/exclusion criteria uniformity	Sample size sufficient and/or described	Exposure measured prior to outcome	Sufficient time frame for association between exposure and outcome	Inclusion exposure level	Exposure measures valid and reliable	Multiple exposure measurements	Outcome measures valid and reliable	Outcome assessor(s) blinded	Loss to follow up post‐baseline 20% or less	Confounders measured and adjusted statistically between exposure and outcome	Quality rating*
Barus et al. (2023)	Yes	Yes	Yes	NR	Yes	No	Yes	Yes	Yes	Yes	Yes	Yes	Yes	Yes	Good
Abdul‐Sattar, Abou, et al. (2014)	Yes	Yes	Yes	Yes	No	No	No	Yes	Yes	No	No	No	No	Yes	Fair
Abdul‐Sattar, Elewa, et al. (2014)	Yes	Yes	Yes	Yes	No	No	No	No	Yes	No	Yes	No	No	Yes	Fair
Akbarzadeh et al. (2021)	Yes	Yes	NR	Yes	No	No	No	Yes	Yes	No	Yes	No	No	No	Poor
Anthony et al. (2011)	Yes	Yes	Yes	NR	No	No	No	Yes	Yes	No	Yes	Yes	No	Yes	Fair
Anttila et al. (1999)	Yes	Yes	Yes	Yes	No	No	No	Yes	Yes	No	Yes	No	No	No	Fair
Arruda and Bigal (2012)	Yes	Yes	Yes	Yes	No	No	No	Yes	Yes	No	Yes	Yes	No	No	Fair
Arruda et al. (2010)	Yes	Yes	Yes	Yes	No	No	No	Yes	Yes	No	Yes	Yes	No	Yes	Fair
Baiden et al. (2021)	Yes	Yes	Yes	Yes	No	No	No	No	Yes	No	Yes	Yes	No	Yes	Fair
Beeckman, Hughes, et al. (2019)	No	Yes	NR	Yes	No	No	No	Yes	Yes	No	Yes	No	No	Yes	Poor
Bener et al. (2000)	Yes	Yes	Yes	Yes	Yes	No	No	NR	NR	No	Yes	No	No	Yes	Fair
Beveridge et al. (2018)	Yes	Yes	Yes	Yes	No	No	No	Yes	Yes	No	Yes	Yes	No	Yes	Fair
Bode et al. (2003)	Yes	Yes	Yes	Yes	No	No	No	No	No	No	Yes	Yes	No	Yes	Fair
Boey et al. (2000)	Yes	Yes	NR	Yes	No	No	No	No	Yes	No	Yes	Yes	No	No	Poor
Boey and Goh (2001a)	Yes	Yes	Yes	Yes	No	No	No	No	No	No	Yes	Yes	No	No	Poor
Boey and Goh (2001b)	Yes	Yes	Yes	Yes	No	No	No	No	Yes	No	Yes	No	No	No	Poor
Chaney and Peterson (1989)	Yes	Yes	Yes	Yes	No	No	No	Yes	Yes	No	Yes	No	No	No	Fair
Connelly et al. (2010)	Yes	Yes	Yes	Yes	Yes	No	Yes	Yes	Yes	Yes	Yes	Yes	No	Yes	Good
Connelly et al. (2012)	Yes	Yes	Yes	Yes	Yes	No	No	Yes	Yes	Yes	Yes	Yes	No	Yes	Good
Cordts et al. (2016)	Yes	Yes	NR	Yes	No	No	No	Yes	Yes	No	Yes	Yes	No	Yes	Fair
Cuneo and Schiaffino (2002)	Yes	Yes	No	Yes	No	No	No	Yes	No	No	No	Yes	No	Yes	Poor
Cunningham et al. (2014)	Yes	Yes	Yes	Yes	No	No	No	Yes	Yes	No	Yes	Yes	No	No	Fair
Dupen et al. (2016)	Yes	Yes	NR	Yes	No	No	No	Yes	Yes	No	Yes	No	No	No	Poor
Eidlitz‐Markus et al. (2015)	Yes	Yes	No	Yes	No	No	No	Yes	Yes	No	Yes	Yes	No	No	Fair
Eidlitz‐Markus and Zeharia (2017)	Yes	Yes	Yes	Yes	Yes	No	No	No	Yes	No	Yes	Yes	No	No	Fair
Evans, Meldrum, et al. (2010)	Yes	Yes	NR	Yes	No	No	No	Yes	Yes	No	Yes	Yes	No	No	Fair
Evans, Taub, et al. (2010)	Yes	Yes	NR	Yes	No	No	No	No	Yes	No	Yes	No	No	Yes	Poor
Feinstein et al. (2018)	Yes	Yes	Yes	Yes	No	No	No	Yes	Yes	No	Yes	Yes	No	Yes	Fair
Feldman et al. (2010)	Yes	Yes	NR	Yes	Yes	No	No	Yes	Yes	No	Yes	No	No	Yes	Fair
Frare et al. (2002)	Yes	Yes	NR	Yes	No	No	No	Yes	No	No	No	No	No	No	Poor
Fryer et al. (2017)	Yes	Yes	NR	Yes	Yes	No	No	No	No	No	Yes	No	No	Yes	Poor
Fuh et al. (2010)	Yes	Yes	NR	Yes	No	No	No	Yes	Yes	No	Yes	No	No	Yes	Fair
Galli et al. (2009)	Yes	Yes	Yes	Yes	No	No	No	No	No	No	Yes	Yes	No	No	Poor
Gaultney et al. (2017)	Yes	Yes	NR	NR	No	No	No	Yes	Yes	Yes	Yes	Yes	No	Yes	Fair
Ghandour et al. (2004)	Yes	Yes	Yes	Yes	Yes	No	No	Yes	Yes	No	Yes	Yes	No	Yes	Fair
Gmuca et al. (2019)	Yes	Yes	NR	Yes	No	No	No	Yes	Yes	No	Yes	Yes	No	No	Fair
Grasaas et al. (2023)	Yes	Yes	Yes	Yes	Yes	No	No	Yes	Yes	No	Yes	No	No	Yes	
Graungaard et al. (2016)	Yes	Yes	No	Yes	No	No	No	Yes	No	No	Yes	Yes	No	Yes	Fair
Groenwald et al. (2020)	Yes	Yes	Yes	Yes	No	No	No	No	No	No	No	Yes	No	No	Poor
Hechler et al. (2011)	Yes	Yes	Yes	Yes	No	No	No	Yes	Yes	No	Yes	Yes	No	Yes	Fair
Hoftun et al. (2013)	Yes	Yes	Yes	Yes	No	No	No	Yes	Yes	No	Yes	Yes	No	Yes	Fair
Holstein et al. (2018)	Yes	Yes	Yes	Yes	Yes	No	No	Yes	Yes	No	Yes	No	No	Yes	Fair
Işik et al. (2009)	Yes	Yes	Yes	Yes	Yes	No	No	Yes	Yes	No	Yes	No	No	Yes	Fair
Kaczynski et al. (2009)	Yes	Yes	Yes	Yes	No	No	No	Yes	Yes	No	Yes	No	No	Yes	Fair
Kaczynski et al. (2013)	Yes	Yes	Yes	Yes	No	No	No	Yes	Yes	No	Yes	Yes	No	Yes	Fair
Kaczynski et al. (2016)	Yes	Yes	Yes	Yes	No	No	No	Yes	Yes	No	Yes	Yes	No	Yes	Fair
Kristjánsdóttir (1996)	Yes	Yes	Yes	Yes	Yes	No	No	Yes	Yes	No	Yes	No	No	No	Fair
Kristjánsdóttir and Wahlberg (1993)	Yes	Yes	Yes	Yes	Yes	No	No	Yes	Yes	No	Yes	No	No	No	Fair
Kröner‐Herwig et al. (2008)	Yes	Yes	Yes	Yes	Yes	No	No	Yes	No	No	Yes	No	No	Yes	Fair
Langer et al. (2011)	Yes	Yes	NR	NR	No	No	No	Yes	Yes	No	Yes	Yes	No	Yes	Fair
Langer et al. (2014)	Yes	Yes	NR	NR	No	No	No	Yes	Yes	No	Yes	Yes	No	No	Poor
Lewandowski and Palermo 2009	Yes	Yes	No	Yes	No	No	No	Yes	Yes	No	Yes	NR	No	Yes	Fair
Lindley et al. 2005	Yes	Yes	NR	Yes	No	Yes	Yes	NR	No	No	NR	Yes	No	No	Poor
Logan and Scharff (2005)	Yes	Yes	Yes	Yes	No	No	No	Yes	Yes	No	No	Yes	No	Yes	Fair
Lynch‐Jordan et al. (2018)	Yes	Yes	NR	Yes	No	No	No	Yes	Yes	No	No	Yes	No	Yes	Fair
Lynch‐Jordan et al. (2013)	Yes	Yes	Yes	Yes	No	No	No	Yes	Yes	No	Yes	Yes	No	No	Fair
Mansuri et al. (2020)	Yes	Yes	Yes	Yes	Yes	No	No	Yes	Yes	No	Yes	No	No	Yes	Fair
Marmorstein et al. (2009)	Yes	Yes	Yes	Yes	No	No	No	No	Yes	No	No	No	No	Yes	Poor
Miró et al. (2019)	Yes	Yes	Yes	No	No	No	No	Yes	Yes	No	Yes	No	No	Yes	Fair
Moore et al. (2019)	Yes	Yes	NR	Yes	No	Yes	Yes	Yes	Yes	No	Yes	No	NR	Yes	Fair
Nelson et al. (2018)	Yes	Yes	No	Yes	No	No	No	Yes	Yes	No	Yes	No	No	Yes	Fair
Stensland et al. (2014)	Yes	Yes	Yes	Yes	No	No	No	Yes	Yes	No	Yes	No	No	Yes	Fair
Oswari et al. (2019)	Yes	Yes	Yes	Yes	Yes	No	No	Yes	No	No	Yes	No	No	Yes	Fair
Robins et al. (2005)	Yes	Yes	NR	Yes	No	No	No	Yes	Yes	No	Yes	Yes	No	No	Fair
Roman‐Juan et al. (2023)	Yes	Yes	NR	Yes	No	No	No	Yes	Yes	No	Yes	Yes	No	No	Fair
Roman‐Juan et al. (2024)	Yes	Yes	NR	Yes	No	No	No	Yes	Yes	No	Yes	Yes	No	No	Fair
Schanberg et al. (2021)	Yes	Yes	Yes	Yes	No	No	No	Yes	Yes	No	Yes	Yes	No	No	Fair
Shaygan and Karami (2020)	Yes	Yes	Yes	Yes	Yes	No	No	Yes	Yes	No	Yes	Yes	No	Yes	Fair
Sieberg et al. (2011)	Yes	Yes	Yes	Yes	No	No	No	Yes	Yes	No	Yes	Yes	No	Yes	Fair
Simons et al. (2008)	Yes	Yes	Yes	Yes	No	No	No	Yes	Yes	No	Yes	Yes	No	Yes	Fair
Siu et al. (2012)	Yes	Yes	Yes	Yes	No	No	No	Yes	Yes	No	Yes	No	No	Yes	Fair
Solé et al. (2024)	Yes	Yes	Yes	Yes	No	Yes	Yes	Yes	Yes	No	Yes	No	NR	Yes	Fair
Stone et al. (2018)	Yes	Yes	Yes	Yes	No	No	No	Yes	Yes	No	Yes	Yes	No	Yes	Fair
Tavasoli et al. (2013)	Yes	No	NR	Yes	No	No	No	No	No	No	Yes	Yes	No	No	Poor
Timmers et al. (2019)	Yes	Yes	NR	Yes	No	No	No	Yes	Yes	No	Yes	Yes	No	No	Fair
Unalp et al. (2007)	Yes	Yes	NR	Yes	Yes	No	No	No	No	No	Yes	No	No	No	Poor
Verstappen et al. (2015)	Yes	Yes	NR	NR	No	No	No	Yes	Yes	No	Yes	Yes	No	No	Poor
Voerman et al. (2015)	Yes	Yes	Yes	Yes	No	No	No	No	Yes	No	Yes	No	No	Yes	Fair
Vuorimaa et al. (2011)	Yes	Yes	Yes	Yes	No	No	No	Yes	Yes	No	Yes	Yes	No	Yes	Fair
Wager et al. (2020)	Yes	Yes	Yes	Yes	No	No	No	No	Yes	No	Yes	No	No	Yes	Fair
Williams et al. (2017)	Yes	Yes	Yes	Yes	No	No	No	Yes	Yes	No	Yes	Yes	No	Yes	Fair

Abbreviation: NR, not reported.

* Quality Rating: Poor: 0–6; Fair: 7–10; and Good: 11–14.

## 3 Results

### 3.1 Citation Search and Selection of Studies

**FIGURE 1:**
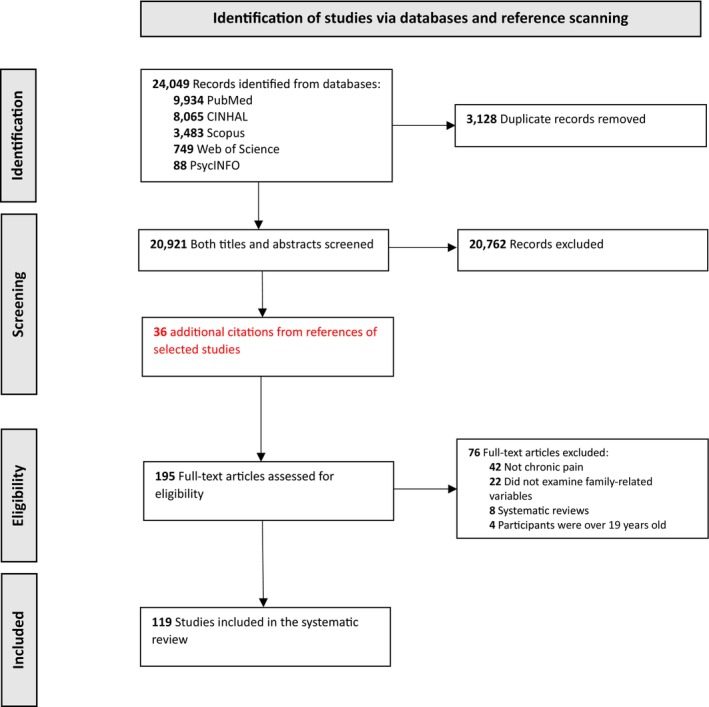
Flow chart showing the process followed to identify the studies included in the review in accordance with PRISMA guidelines.

The initial search retrieved 24,049 citations, of which 3128 were duplicated, so 20,921 were screened by title and abstract. Forty additional citations were identified by scanning the references of selected studies. A total of 195 articles were selected for a full‐text review, of which 76 were excluded for not meeting the inclusion criteria. As a result, a total of 119 articles were read in full for this review. The article selection process is shown in Figure 1. The findings of the studies included in this review are summarised in Table 4.

**TABLE 4:** Characteristics and findings of the studies reviewed.

Author/s and year	Sample (*N*, *M*_age_, age range) and type of pain	Study design	Family factors included	Questionnaires used to assess family factors	Child pain‐related outcomes	Measures of child pain‐related outcomes	Study outcomes
Aasland et al. (1997)	*N* = 23, *M*_age_ = 12 (range: 5–15), Idiopathic Musculoskeletal Pain; *N* = 52, *M*_age_ = 9 (range: 1–16), JIA	Longitudinal	(1) Mothers' and fathers' education; (2) Family structure; (3) Family history of musculoskeletal limiting pain; (4) Employment (5) Education (6) Economy, (7) Housing conditions, (8) Marital or family discord, (9) Social network and (10) Physical and mental health; (11) Presence and impact of family stressful life events	(1–10) Semi structured interview; (11) Recent Life Change Questionnaire	(1) Persistence of idiopathic chronic musculoskeletal pain	Presence of idiopathic chronic musculoskeletal pain at 9‐year follow‐up	Mothers' education was negatively associated with persistence of idiopathic chronic musculoskeletal pain. Family history of idiopathic musculoskeletal pain and chronic family difficulties were positively associated with persistence of idiopathic chronic musculoskeletal pain
Barus et al. (2023)	*N* = 3605, *M*_age_ = 16.2 (range: 15–19), Headache	Cross‐sectional	(1) Economic status	(1) Survey questionnaire	(1) Educational level; (2) Economic status; (3) Smoking; (4) Depression	(1–3) Survey questionnaire; (4) Center of Epidemiologic Studies Depression Scale (10‐ item CES‐D Scale)	Low socioeconomic status (SES) showed a positive association with headaches
Abdul‐Sattar, Abou, et al. (2014)	*N* = 58, *M*_age_ = 9.5 (range: 7–17), JIA	Cross‐sectional	(1) Rural residency; (2) Family income per month; (3) Mothers' illiteracy	(1, 2, 3) Study questionnaire	(1) School attendance; (2) School functioning	(1) Number of missed days due to JIA over the previous academic year; (2) Paediatric Quality of Life Inventory 4.0 generic core scales	Living in a rural area was an independent predictor of school absenteeism in children with JIA
Abdul‐Sattar, Elewa, et al. (2014)	*N* = 58, *M*_age_ = 9.1 (range: 7–17), JIA	Cross‐sectional	(1) Rural residency; (2) Low or very low socioeconomic status	(1, 2) Study questionnaire	(1) Health related quality of life	(1) Paediatric Quality of life Inventory 4.0 generic core Scales	Low socioeconomic status was independently associated with impaired health‐related quality of life in children with JIA
Akbarzadeh et al. (2021)	*N* = 132, *M*_age_ = 9.83 (range: 7–16), JIA	Cross‐sectional	(1) Parental pain catastrophizing	(1) Pain Catastrophizing Scale Parent Version	(1) Anxiety; (2) Depression; (3) Pain intensity	(1–2) Child Behavioural Check List; (3) 0–10 Numerical rating scale	Significant association between maternal pain catastrophizing and estimates of pain intensity by mothers
Anthony et al. (2011)	*N* = 51, *M*_age_ = 12.4 (range: 8–16), Juvenile polyarticular arthritis	Cross‐sectional	(1) Depressive symptoms; (2) Parental perceptions of child vulnerability; (3) Stress	(1) Beck Depression Inventory–Second Edition; (2) Child Vulnerability Scale; (3) Hassles and Uplifts Scale	(1) Depressive symptoms; (2) Anxiety; (3) Pain intensity	(1) Children's Depression Inventory; (2) Revised Children's Manifest Anxiety Scale; (3) Visual analogue scale	Higher parental perceptions of child vulnerability predicted increased child depressive symptoms and anxiety. Parent stress predicted both child anxiety and pain
Aromaa et al. (1998)	*N* = 968, *M*_age_ = 6 (range: 1–6), Headache	Longitudinal	(1) Mother's prepregnancy headache; (2) Mother's proteinuria during pregnancy; (3) Other family member suffering from headache	(1) Face‐to‐face interviews with the practitioner	(1) Headache disturbing daily activities during the previous 6 months	(1) Study questionnaire	Mother's report of the infant's poor health and feeding problems predicted later occurrence of headache. At 3 years, depression and sleeping difficulties and recurrent difficulties in falling asleep were strong predictors of headache. Headache in other family members, especially in the mother, predicted preschool headache in the child
Anttila et al. (1999)	*N* = 1433, *M*_age_ = NR (range: 6–7), Headache	Cross‐sectional	(1) Family happiness; (2) Paroxysmal headache in a family member; (3) Poor economic situation; (4) Unemployment in a family member	(1–4) Study questionnaire	(1) Occurrence of migraine	(1) Study questionnaire	The occurrence of familial paroxysmal headache and unhappiness in the family independently predicted the occurrence of migraine in children
Arruda and Bigal (2012)	*N* = 1856, *M*_age_ = NR (range: 5–12), Headache	Cross‐sectional	(1) Race; (2) Income class; (3) Headache status; (4) Headache frequency in the mother	(1–4) Study's standardised interview	(1) Presence of chronic migraine; (2) Behavioural and emotional functioning	(1) Study's standardised interview; (2) Child Behaviour Checklist (CBCL)	Prevalence of migraine with and without aura was significantly higher in lower income classes. Children with migraine had significantly different CBCL scores relative to children without migraine and this did not vary significantly as a function of headache status in the mother
Arruda et al. (2010)	*N* = 1326, *M*_age_ = 7.9 (range: 5–12), Headache	Cross‐sectional	(1) Frequency of headaches in the mother; (2) Socioeconomic status	(1–2) Study's standardised interview	(1) Frequency of headache	(1) Study's standardised questionnaire	Frequency of headaches in children was influenced by frequency of headaches in the mother. Low socioeconomic status independently predicted child headache frequency
Baiden et al. (2021)	*N* = 40,953, *M*_age_ = 10.0 (range: 3–17), Headache	Cross‐sectional	(1) Exposure to adverse childhood experiences; (2) Household poverty level	(1) Study's standardised questionnaire	(1) Presence of frequent or severe headaches	(1) Study's standardised questionnaire	Adverse childhood experiences were positively associated with frequent or severe headaches
Beeckman, Hughes, et al. (2019)	*N* = 59, *M*_age_ = 13.76 (range: 8–18), JIA	Cross‐sectional	(1) General psychological flexibility; (2) Child pain‐specific psychological flexibility	(1) Acceptance and Action Questionnaire; (2) Parental Psychological Flexibility Questionnaire	(1) Psychosocial functioning (PF); (2) Disability; (3) Positive affect; (4) Negative affect	(1) The Generic Core version of the Paediatric Quality of Life Inventory; (2) Study's standardised questionnaire; (3) Positive and Negative Affect Scale for Children	Parental general PF indirectly contributed to child psychosocial functioning and affect via the child's general PF. Parent pain‐specific PF was indirectly associated with child psychosocial functioning, disability, and negative affect via child pain acceptance
Beeckman, Simons, et al. (2019)	*N* = 56, *M*_age_ = 14.50 (range: 11–17), Chronic pain	Longitudinal	(1) Parent protective responses; (2) Parent engagement instructions	(1) Inventory of Parent/Caregiver Reponses to the Children's Pain Experience; (2) Study's standardised questionnaire	(1) Pain intensity; (2) Activity avoidance; (3) Activity engagement	(1) 0–10 Numerical rating scale; (2) Avoidance of Activities' subscale of the Fear of Pain Questionnaire for Children	Psychologically flexible parenting and acceptance of adolescent pain in parents were indirectly associated with lower daily adolescent activity‐avoidance. Psychologically flexible parenting and parental acceptance of adolescent pain were also indirectly associated with daily decreases in adolescent activity‐avoidance
Bener et al. (2000)	*N* = 1159, *M*_age_ = NR (range: 6–14), Headache	Cross‐sectional	(1) Family history of migraine; (2) Being unhappy at home	(1–2) Study questionnaire—child reported	(1) Presence of migraine	(1) Study questionnaire—child reported	Family history of migraine and being unhappy at home were significant predictors of headache in children and adolescents after adjusting for age and sex
Beveridge et al. (2018)	*N* = 173, *M*_age_ = 13.43 (range: 8–18), Chronic pain	Cross‐sectional	(1) Parent post‐traumatic stress disorder (PTSD) symptoms	(1) Parent PTSD symptoms Checklist for DSM‐5 (PCL‐5)	(1) Post‐traumatic stress disorder symptoms; (2) Pain interference; (3) Pain intensity; (4) Pain duration; (5) Health‐related quality of life	(1) Child PTSD Symptom Scale; (2) Pain interference subscale of the Patient‐Reported Outcomes Measurement Information System (PROMIS)‐25 Profile; (3) 0–10 Numerical Rating Scale (4) Pain Questionnaire; (5) Paediatric Quality of Life Short Form	Having a parent with chronic pain may confer additional risk for children with chronic pain experiencing higher post‐traumatic stress disorder symptoms, worst pain outcomes, and lower health‐related quality of life
Beveridge et al. (2020)	*N* = 3914, *M*_age_ = (range: 10–18), Chronic pain	Case–control	(1) Adverse childhood events (ACEs)	(1) Adverse childhood events questionnaire	(1) Child ongoing chronic pain	(1) Clinical assessment	Controlling for sociodemographic factors, ACEs were similar across samples, except that parents of young people with chronic pain reported significantly higher rates of physical neglect than the community‐based non‐chronic sample
Bizzi et al. (2020)	*N* = 94, *M*_age_ = 12.77 (range: 10–14), Headache	Case–control	(1) Emotional openness; (2) Balance of positive and negative reference to attachment figures; (3) Preoccupied anger to mother; (4) Preoccupied anger to father; (5) Idealisation to mother; (6) Idealisation to father; (7) Dismissal to mother; (8) Dismissal to father; (9) Resolution of conflicts; (10) Overall coherence; (11) Reflective functioning	(1–11) Child Attachment Interview; (11) Child and Adolescent Reflective Functioning Scale	(1) Child primary headache	(1) Clinical assessment	Adolescents with primary headache showed a greater percentage of insecure‐preoccupied attachment to both parents, with a higher level of preoccupied anger especially to father
Bode et al. (2003)	*N* = 1522, *M*_age_ = NR (range: 5–8), RAP	Cross‐sectional case‐controlled study	(1) Family demographics; (2) Socioeconomic status; (3) Housing and living conditions; (4) Education of parents; (5) Single parent household; (6) Number of siblings; (7) History of gastrointestinal disorders other than ulcer of mother or father; and (8) Smoking in the household	Structured questionnaire and C‐urea breath test which indicates current infection with *H. pylori*	(1) Child primary abdominal pain	(1) Study's standardised questionnaire	Social and familial factors like living in a single parent household or history of gastrointestinal disorders within the family play a major role in the development/maintenance of RAP, but not *Helicobacter pylori* infection
Boey et al. (2000)	*N* = 1549, *M*_age_ = NR (range: 11–16), RAP	Cross‐sectional	(1) Number of siblings; (2) School location; (3) Ethnic origin; (4) Father's highest educational attainment; (5) Family income.	(1–3) Study questionnaire—parents report	(1) Presence of RAP interfering with normal daily activity	(1) Child self‐reported	There are significant differences in the prevalence of RAP between children from rural and urban schools, among children with different family incomes and among children whose parents have different educational backgrounds
Boey and Goh (2001a)	*N* = 1488, *M*_age_ = NR (range: 9–15), RAP	Cross‐sectional	(1) Ethnic group; (2) Fathers' occupation; (3) Fathers' educational level; (4) Family history of chronic abdominal pain; (5) Siblings with RAP.	(1–3) Study questionnaire—parents report	(1) Presence of RAP interfering with normal daily activity	(1) Child self‐reported	The results of the study showed that RAP was associated with a number of demographic variables, a history of abdominal complaints in the family, father without employment and a low educational level
Boey and Goh (2001b)	*N* = 1488, *M*_age_ = NR, (range: 9–15), RAP	Cross‐sectional	(1) Life‐events	(1) Child self‐reported	(1) Presence of RAP interfering with normal daily activity	(1) Child self‐reported	Recent stressful life‐events are important risk‐factors for RAP
Chaney and Peterson (1989)	*N* = 25, *M*_age_ = 12.5 (range: 7–17), Rheumatoid arthritis	Cross‐sectional	(1) Knowledge of medication regimen; (2) Family adaptability and cohesion; (3) Family satisfaction; (4) Family coping; (5) Family life events	(1) Medical quiz; (2) Family Adaptability and Cohesion Evaluation Scales III; (3) Family Satisfaction questionnaire; (4) Family Crisis Oriented Personal Evaluation Scales; (5) The Family Inventory of Life Events and Changes	(1) Disease activity; (2) Medication compliance	(1) Clinical assessment; (2) Parent reported, weekly medication diary	Strong and significant association across several measures of family functioning and a self‐monitoring index of medication compliance. Family functioning was not related to disease activity
Chow et al. (2016)	*N* = 195, *M*_age_ = 13.8 (range: 8–19), Chronic pain	Longitudinal	(1) Parent pain catastrophizing; (2) Parent fear of pain; (3) Parent protective behaviour	(1) Pain Catastrophizing Scale for parents; (2) Parent Fear of Pain Questionnaire; (3) Adult Responses to Children's Symptoms	(1) Pain catastrophizing; (2) Fear of pain; (3) Anxiety; (4) Depression; (5) Functional disability; (6) School Functioning	(1) Pain Catastrophizing Scale for children; (2) Fear of Pain Questionnaire for children; (3) Revised Children's Manifest Anxiety Scale; (4) Children's Depression Inventory short form; (5) Functional Disability Index; (6) Paediatric Quality of Life Inventory	Parent distress and behaviour influenced child distress and functioning over time
Clementi et al. (2019)	*N* = 205, *M*_age_ = 13.84 (range: 10–17), Chronic musculoskeletal pain	Case–control	(1) Child ethnicity; (2) Child race; (3) Parent ethnicity; (4) Parent race; (5) Parent education; (6) Household income; (7) Parent current chronic pain; (8) Parent somatization; (9) Protective parenting behaviour	(1–6) Standardised questionnaire; (7) Single self‐report item; (8) Somatization subscale of the Brief Symptom Inventory‐18; (9) Protective subscale from Adult Responses to Children's Symptoms	(1) Presence of chronic musculoskeletal pain	(1) Diagnosis of chronic musculoskeletal pain in the limb(s), back, or neck with pain present for 3 months or longer, pain occurring at least weekly, and pain‐related functional disability	Parents of youth with acute and chronic pain had higher rates of chronic pain than parents of youth without pain. Protective parenting behaviours did not significantly differ by group. The presence of current parental chronic pain and greater parent protective behaviours were associated with higher child‐reported acute pain intensity
Coenders et al. (2014)	*N* = 101, *M*_age_ = 14.1 (range: 10–18), Chronic pain	Case–control	(1) Parental history of growing pains; (2) Parental history of migraine; (3) Parental history of non‐migraine headaches; (4) Parental history of RAP; (5) Parental history of restless leg syndrome; (6) Parental history of fibromyalgia; (7) Parental depression; (8) Parental anxiety; (9) Parental stress	(1–6) Standardised self‐administered questionnaire; (7–9) Depression Anxiety Stress Scale.	(1) Presence of chronic pain	(1) Standardised self‐report questionnaire	Parental migraine, RAP, and restless leg syndrome were significantly associated with adolescent chronic pain. Individual history of migraine, non‐migraine headaches, and RAP, along with parental history of RAP and depression significantly accounted for 36%–49% of variance in chronic pain
Connelly et al. (2010)	*N* = 9, *M*_age_ = 12.3 (range: 8–16), JIA	Cross‐sectional	(1) Mood; (2) Caregiver responses to child pain	(1) Positive and Negative Affect Schedule; (2) Adult Responses to Children's symptoms Questionnaire	(1) Pain; (2) Mood, (3) Activity Interference	(1) Visual Analogue Scale; (2) Positive and Negative Affect Schedule for Children; (3) Child Activity Limitations Questionnaire	Use of protective pain responses by parents significantly predicted decreases in child activity and positive mood, with an even stronger inverse relationship between protective pain response and positive mood observed in children with higher than average disease severity. Distracting responses significantly predicted less child activity restrictions but only in children with higher disease severity and tended to be associated with lower child positive mood in children with JIA
Connelly et al. (2012)	*N* = 87, *M*_age_ = 13.5 (range: 6–18), Chronic pain	Cross‐sectional	(1) Caregiver distress; (2) Perceived Child Vulnerabilities	(1) Brief Symptom Inventory); (2) Child Vulnerability Scale	(1) Child functioning; (2) Health care utilisation	(1) Paediatric Quality of Life Inventory; (2) Parent proxy report measure	Parent perceptions of greater child vulnerability were significantly associated with poorer child functioning and greater use of child pain‐related health care regardless of the child's age, sex, and duration of pain. Parent distress was found to be indirectly associated with the child's health care utilisation through parent perceptions of child vulnerability but directly related to child functioning
Conte et al. (2003)	*N* = 48, *M*_age_ = 13.60 (range: 9–17), Juvenile primary fibromyalgia or arthritis	Case–control	(1) Family environment; (2) Pain sensitivity; (3) Parents psychological adjustment	(1) Family environment scale‐child and parent report; (2) Sensitivity Temperament Inventory for Pain; (3) Symptom Checklist‐90‐Revised	(1) Presence of Juvenile Primary Fibromyalgia; (2) Presence of Arthritis	(1–2) Clinical diagnosis	Parents of children with juvenile primary fibromyalgia syndrome, self‐reported higher levels of anxiety and depression, and lower overall psychological adjustment compared with parents of children in the arthritis and healthy groups
Cordts et al. (2016)	*N* = 146, *M*_age_ = 12.97 (range: 8–18), Chronic pain	Cross‐sectional	(1) Parental number of chronic pain locations; (2) Parental pain intensity; (3) Parental pain interference; (4) Parental physical functioning; (5) Parental protectiveness; (6) Parental monitoring; (7) Parental catastrophizing; (8) Parental anxiety; (9) Parental depressive symptoms.	(1–2) Self‐reported Pain Questionnaire; (3–4, 8–9) PROMIS‐29 Profile; (5–6) Adult Responses to Children's Symptoms; (7) Pain Catastrophizing Scale‐Parent Version.	(1) Number of chronic pain locations; (2) Pain intensity; (3) Pain interference; (4) Anxiety; (5) Depressive symptoms; (6) Catastrophizing	(1–2) Self‐reported Pain Questionnaire; (3–5) PROMIS‐25 Profile; (6) Pain Catastrophizing Scale‐ Child Version	Parent pain‐related characteristics and psychosocial functioning were associated with child pain and pain interference and psychosocial functioning variables. Parent protectiveness and monitoring were not associated with child pain and pain‐related interference
Crushell et al. (2003)	*N* = 28, *M*_age_ = NR (range: 1–6), RAP	Longitudinal	(1) Parental conceptual model of illness	(1) Telephone interview	(1) Persistence/remission of RAP	(1) Clinician diagnoses	The acceptance by parents of a biopsychosocial model of illness was associated with the remission of RAP symptoms in children
Cuneo and Schiaffino (2002)	*N* = 57, *M*_age_ = 14.26 (range: 11–18), Arthritis	Cross‐sectional	(1) Family cohesion; (2) Family conflict; (3) Social support from family and friends; (4) Parental depression; (5) Parental self‐ worth	(1–2) Family Environmental Scale; (3) Perceived Social Support from Friends; (3) Perceived Social Support from Family; (4) Beck Depression Inventory; (5) Adult Self‐Perception Profile.	(1) Adolescent Self‐worth; (2) Child Depression	(1) Self‐Perception Profile for Adolescents; (2) Children's Depression Inventory	Family functioning was significantly associated with adolescent adjustment across different conceptualizations and measures of adjustment
Cunningham et al. (2014)	*N* = 75, *M*_age_ = 13.84 (range: 9–18), RAP	Cross‐sectional	(1) Parental protection; (2) Parental minimization; (3) Parental encouraging/monitoring response; (4) Parent pain catastrophizing	(1–3) Adult Responses to Children's Symptoms; (4) Pain Catastrophizing Scales Parent Version	(1) Pain intensity; (2) Pain catastrophizing; (3) Functional disability	(1) 0–10 Numerical Rating Scale; (2) Pain Catastrophizing Scales Child Version; (3) Functional Disability Inventory‐child report	The impact of parenting behaviours in response to functional abdominal pain on child disability was determined in part by the child's coping style
Dougherty et al. (2021)	*N* = 225 *M*_age_ = 13.95 (range: 8–17), Chronic musculoskeletal pain	Longitudinal	(1) Race; (2) Ethnicity; (3) Parent catastrophizing; (4) Parent protective behaviours	(1–2) Study's questionnaire (3) The Pain Catastrophizing Scales—Parent version; (4) Adult Responses to Children's Symptoms	(1) Child functional disability; (2) Pain catastrophizing	(1) Functional disability inventory; (2) The Pain Catastrophizing Scales—Child version	Greater engagement in protective behaviours leads to a slower improvement in the child's functional disability. Higher levels of parental catastrophizing lead to higher levels of child catastrophizing and higher levels of parent‐reported child functional disability
Dupen et al. (2016)	*N* = 316, *M*_age_ = 9.4 (range: 7–12), Functional recurrent abdominal pain disorder	Cross‐sectional	(1) Protective behaviours	(1) Adult Responses to Children's Symptoms Scale	(1) Gastrointestinal severity; (2) Child functional disability (parent‐report)	(1) Children's Somatization Inventory (parent‐report); (2) Functional Disability Inventory (parent‐report)	Parents who perceived their child to have low self‐efficacy to cope with pain responded more protectively when they believed the child was in pain, and this was associated with higher levels of gastrointestinal symptoms and disability in their child
Eidlitz‐Markus et al. (2015)	*N* = 344, *M*_age_ = 11.69 (range: 3–18), Migraine	Cross‐sectional	(1) Family history of migraine	(1) International Headache Society‐based questionnaire	(1) Age of onset of migraine; (2) Duration of headache attacks; (3) Number of headache attacks per month	(1–3) International Headache Society‐based questionnaire	The age of migraine onset of children with a family history of migraine was significantly lower than in children with no family history of migraine. Duration of headache episodes was significantly higher among children with a family history of migraine than among children without a family history of migraine
Eidlitz‐Markus and Zeharia (2017)	*N* = 102, *M*_age_ = 12.6 (range: 4–18), Migraine	Cross‐sectional	(1) History of migraine	(1) Questionnaire based on the International Classification of Headache Disorders	(1) Age at migraine onset; (2) Headache frequency per month; (3) Duration of headache attacks	(1–3) Questionnaire based on the International Classification of Headache Disorders	Children with migraine were significantly younger at first appearance of the disease than their affected parents
Ertem et al. (2019)	*N* = 238, *M*_age_ = 12.59 (range 9–16), Migraine	Case–control	(1) Parental attitudes	(1) Parental Attitudes Determining Scale	(1) Anxiety; (2) Depression	(1) Anxiety Scale for Adolescents; (2) Children's Depression Inventory	Mean oppressive‐authoritarian attitude subscale scores of mothers was significantly higher in children with chronic migraine
Esposito et al. (2013)	*N* = 856, *M*_age_ = 8.74 (range: 6–12), Migraine	Case–control	(1) Mothers' personality traits	(1) Minnesota Multiphasic Personality Inventory second editionMMPI‐2	(1) Migraine without aura (MoA) characteristics: intensity, frequency, and duration of migraine attacks	(1) Clinical assessment	Mothers of children with MoA showed significantly higher scores in paranoia and social introversion, anxiety, obsessiveness, depression, health concerns, bizarre mentation, cynicism, type A, low self‐esteem, and work interference. A significant relationship was found between MoA frequency of children and anxiety and low self‐esteem. MoA duration of children was related with hypochondriasis, hysteria, paranoia, psychasthenia, schizophrenia, anxiety, and health concerns in the MMPI‐2 scores of their mothers
Evans, Meldrum, et al. (2010)	*N* = 179, *M*_age_ = 14.34 (range: 11–19), Chronic pain	Cross‐sectional	(1) Mothers' pain symptoms; (2) Parents with chronic pain; (3) Mothers psychological functioning; (4) Mothers' anxiety sensitivity	(1, 2) Demographic information questionnaire developed by the authors; (3) Symptom Checklist‐90R; The Anxiety Sensitivity Index	(1) Somatic problems; (2) Functional Disability; (3) Pain intensity; (4) Anxiety sensibility; (5) Depression	(1) Children's Somatization Inventory)—parent reported; (2) Functional Disability Inventory‐ parent reported; (3) Visual Analogue Scale—mothers reported and child reported; (4) Children's Anxiety Sensitivity Inventory; (5) Child Depression Inventory	The quantitative data suggest stronger mother‐daughter than mother‐son pain associations. The qualitative data suggest that girls' pain and pain‐related disability is associated with an overly enmeshed mother‐daughter relationship and the presence of maternal models of pain, while boys' pain and disability are linked to male pain models and criticism, and to maternal worry and solicitousness
Evans et al. (2018)	*N* = 232, *M*_age_ = 15 (range 12–17), Chronic pain	Case–control	(1) Parental bonding	(1) A modified version of the Parental Bonding Instrument, the Parental Bonding Instrument— Brief Current version	(1) Pain intensity; (2) Depression	(1) 0–10 Numeric Rating Scale; (2) The Revised Children's Anxiety and Depression Scale depression subscale	There were significant associations between parental attachment and adolescent pain and depression in the pain group. There were no differences in the impact of maternal versus paternal bonding on adolescent pain and depression. Adolescent depression was a mediator of the association between maternal care and adolescent pain, and paternal control and adolescent pain
Evans, Taub, et al. (2010)	*N* = 219, *M*_age_ = 14.34 (range: 7–18), Chronic pain	Cross‐sectional	(1) Race/Ethnicity	(1) Patients' medical records	(1) Number and type of pain diagnoses; (2) Duration of pain symptoms; (3) Hours slept; (4) School absenteeism; (5) Type of schooling; (6) Physical and psychosocial well‐being; (7) Psychophysiological symptoms; (8) Functional disability; (9) Pain intensity	(1, 2, 3, 4, 5) Patients' medical records; (6) Child Health Questionnaire, Parent Report; (7) Children's Somatization Inventory—Parent Report; (8) Functional Disability Inventory—Parent report; (9) 0–10 Numeric Rating Scale	Sociodemographic factors were significantly associated with several pain‐related characteristics in children with chronic pain
Feinstein et al. (2018)	*N* = 324, *M*_age_ = 14.72 (range: 10–17), Chronic pain	Cross‐sectional	(1) Pain catastrophizing; (2) Pain acceptance	(1) Pain Catastrophizing Scale for Parents; (2) Chronic Pain Acceptance Questionnaire for Parents	(1) Average pain intensity; (2) Pain interference; (3) Mobility; (4) Pain catastrophizing; (5) Pain acceptance	(1) 0–10 Numeric Rating Scale; (2) PROMIS Pain Interference; (3) PROMIS Mobility; (4) Pain Catastrophizing Scale for Children; (5) Chronic Pain Acceptance Questionnaire for Adolescents	Parent pain catastrophizing and pain acceptance were associated with child function indirectly via child pain catastrophizing and pain acceptance
Feldman et al. (2010)	*N* = 1138, *M*_age_ = NR (range: 5–13), Chronic pain	Cross‐sectional	(1) Parental psychopathology; (2) Parenting style; (3) Coercive discipline‐parent report; (4) Coercive discipline‐child report; (5) Maternal acceptance‐parent report; (6) Acculturative stress‐parent report; (7) Good family functioning‐parent report; (8) Parent–child interaction‐child report; (9) Lifetime neglect‐child report; (10) Lifetime physical abuse‐child report; (11) Verbal/psychological abuse‐child report	(1) Family History Screen for Epidemiologic Studies; (2–3) Parental Monitoring scale; (3–4) The Parental Discipline scale; (5) Maternal Acceptance and Warmth scale; (6) Hispanic Stress Inventory; (7) Family APGAR (adaptability, partnership, growth, affection, and resolve); (8) Parent–Child Involvement scale; (9–11) The Parental Disciplines Practice Scale	(1) Child pain (2) Internalising Disorders	(1) Yes/No study questions (2) The Diagnostic Interview Schedule for Children	Parental psychopathology and acculturative stress were positively associated with childhood abdominal pain, and headaches. Good family functioning was negatively associated with frequent abdominal pain and headaches
Frare et al. (2002)	*N* = 48, *M*_age_ = 11 (range: 8–14), Headache	Cross‐sectional	(1) Family routines	(1) Ecocultural Family Interview	(1) Quality of life	(1) Ecocultural Family Interview	The family daily routine significantly influenced both the child's coping ability and quality of life
Fryer et al. (2017)	*N* = 8463, *M*_age_ = 11 (range: NR), Headache	Cross‐sectional	(1) Maternal education level; (2) Ethnicity; (3) Maternal general health; (4) Maternal gastrointestinal disease; (5) Maternal amount of bodily pain; (6) Maternal distress	(1–5) Study survey; (6) Maternal Kessler scale	(1) Child frequent complaints of pain	(1) Strengths and Difficulties Questionnaire	Children of mothers with no educational qualifications were more likely to have frequent pain complaints than children of mothers with degrees
Fuh et al. (2010)	*N* = 3955, *M*_age_ = 14.0 (range: 13–15), Migraine	Cross‐sectional	(1) Physical maltreatment	(1) 3‐point Likert scale question (study survey)	(1) Headache/migraine; (2) Depression	(1) Survey questionnaire; (2) Assessment of Depression Inventory	Physical maltreatment was associated with migraine in adolescents
Galli et al. (2009)	*N* = 200, *M*_age_ = 11.13 (range: 8–18), Headache	Cross‐sectional	(1) Headache familial recurrence; (2) Pain intensity; (3) Frequency of pain; (4) Psychiatric disorders	(1–4) Study questionnaire	(1) Headache type; (2) Psychiatric comorbidity	(1, 2) Study survey	Children with migraine were characterised by a higher prevalence of psychiatric disorders in parents, co‐occurrence of psychiatric comorbidity and headache familial recurrence than children with non‐migraine headaches
Gaultney et al. (2017)	*N* = 65, *M*_age_ = 12.7 (range: 8–15), JIA	Cross‐sectional	(1) Children pain intensity reported by parents; (2) Children fatigue intensity reported by parents	(1, 2) Visual Analogue Scale	(1) Activity limitations; (2) Negative mood	(1) Activity Scale for Kids and the Child Activity Limitations Questionnaire; (2) Positive and Negative Affect Scale for children	Children reported increased activity limitations and negative mood when parent and child pain ratings were discrepant (with parents rating their child's pain much lower). Greater discrepancy in fatigue ratings was also associated with more negative mood. Children whose parents rated child fatigue as moderately lower than the child experienced decreased activity limitations relative to dyads who agreed more closely on fatigue level
Ghandour et al. (2004)	*N* = 835, *M*_age_ = NR (range:12–15), Headache, Stomachache, Backache	Cross‐sectional	(1) Race/ethnicity; (2) Mothers' education; (3) Parent support	(1, 2, 3) Study survey	(1) Presence of headache, stomachache and backache more than once a week	(1) Study survey	Headache, stomachache and backache were strongly associated with social, environmental, and behavioural risk factors such as perceived social support
Gmuca et al. (2019)	*N* = 28, *M*_age_ = 15.0 (range: 14–15), Chronic musculoskeletal pain	Cross‐sectional	(1) Resilience	(1) CD‐RISC‐10; (1) The RS‐14	(1) Self‐reported health; (2) Anxiety; (3) Depression; (4) Suicidality; (5) Pain intensity; (6) Energy level; (7) Functional disability; (8) Widespread Pain Index; (9) Symptom severity	(1) The PROMIS PGH‐7; (2, 3, 4) medical records; (5) Visual Analogue Scale; (6) verbal pain report; (7) Functional disability inventory; (8, 9) medical records	Parents' resilience was not associated with adolescent outcomes
Grasaas et al. (2023)	*N* = 508, *M*_age_ = 14.09 (range 14–15), Chronic pain	Cross‐sectional	(1) Age (2) Parental marital status (3) Stress (4) Pain	(1–2) sociodemographic questions (3) Perceived Stress Questionnaire (PSQ) (4) Brief pain inventory (BPI)	(1) Age (2) Work status, (3) Educational status, (4) Household income, (5) Stress (6) Pain	(1–4) sociodemographic questions (5) Perceived Stress Questionnaire (PSQ) (6) Brief pain inventory (BPI)	High educational status in mothers and mothers working part‐time were associated with lower pain in Norwegian adolescents
Graungaard et al. (2016)	*N* = 131, *M*_age_ = NR (range: 6–11), Chronic pain	Cross‐sectional	(1) Age; (2) Chronic disease; (3) Chronic pain; (4) Use of analgesics; (5) Daily use of medicine; (6) Self‐rated health	(1–7) Study survey	(1) Recurrent pain	(1) Study survey	Mothers with chronic pain conditions were five times more likely to report pain in their child than mothers without pain
Groenwald et al. (2020)	*N* = 48,567, *M*_age_ = NR (range: 6–17), Chronic pain	Cross‐sectional	(1) Age, (2) Sex; (3) Race and ethnicity; (4) Household Income; (5) Primary language at home	(1–8) Study questionnaire	(1) Anxiety and Depression, (2) Chronic pain, (3) ACEs	(1–3) Study questionnaire	Children exposed to one or more ACEs had higher rates of chronic pain than those with no exposure to ACEs. The strongest associations of individually measured ACEs with chronic pain included financial instability, living with a mentally ill adult, and having experienced race‐based discrimination
Hammond et al. (2019)	*N* = 2313, *M*_age_ = NR (range: 14–15), Migraine	Longitudinal	(1) Hostile/ineffective parenting style; (2) Punitive/aversive discipline; (3) Family dysfunction; (4) Socioeconomic status; (5) Parents' professional‐diagnosed migraine headaches; (6) Parental depressive symptoms	(1, 2) Strayhorn and Weidman's Parent Practices Scale; (3) Family Functioning Scale; (4, 5) Study survey; (6) Centre for Epidemiological Studies Depression scale	(1) Migraine onset; (2) Recurrent headaches onset	(1, 2) Professional diagnoses	Exposure to family dysfunction, punitive parenting, and/or parental depressive symptoms during the first 6 years of life were associated with symptoms of depression and anxiety in late childhood, which, in turn, was associated with migraine incidence at 14–15 years of age
Hechler et al. (2011)	*N* = 128, *M*_age_ = 11.9 (range: 8–17), Chronic Pain	Cross‐sectional	(1) Pain catastrophizing about their child's chronic pain	(1) Parental Pain Catastrophizing Scale	(1) Pain intensity; (2) Pain‐related disability; (3) School absence	(1) 0–10 Numeric Rating Scale; (2) Paediatric Pain Disability Index; (3) Parent report	Maternal but not paternal catastrophizing contributed significantly to explaining the child's pain intensity whereas neither mothers' nor fathers' catastrophizing were significantly associated with the child's disability
Heikkala et al. (2023)	*N* = 5878, Age = 16, Musculoskeletal pain	Case–control	(1) Family structure; (2) Mother's educational level	(1, 2) Survey question	(1) Musculoskeletal pain; (2) Physical activity; (3) Smoking status; (4) Sleeping duration (5) Adolescents emotional and behavioural problems	(1–5) Survey questions	Adolescents living in a single‐parent or reconstructed family have higher odds of multisite musculoskeletal chronic pain than adolescents living in two‐parent families
Helgeland et al. (2010)	*N* = 456, *M*_age_ = NR (range: 1–14), Abdominal pain	Longitudinal	(1) Maternal psychological distress; (2) Maternal physical health; (3) Negative life events; (4) Negative events reported by children	(1) Hopkins Symptom Checklist; (2) Survey question; (3, 4) Survey checklist	(1) Self‐reported RAP	(1) Survey question	Maternal psychological distress in early childhood predicted RAP in their offspring 13 years later
Hoftun et al. (2013)	*N* = 5370, *M*_age_ = NR (range: 13–18), Chronic pain	Cross‐sectional	(1) Family chronic pain; (2) Family structure	(1) Survey question; (2) Statistics Norway	(1) Chronic pain; (2) Multisite chronic pain	(1) Survey questionnaire	Parental chronic pain is associated with chronic nonspecific pain and especially with chronic multisite pain in adolescents and young adults. Family structure influences the association, indicating that family pain models and shared environmental factors are important in the origin of chronic pain
Holstein et al. (2018)	*N* = 31,102, *M*_age_ = NR (range: 11–15), Chronic pain	Cross‐sectional	(1) Parents socioeconomic status	(1) Study survey	(1) Frequent headaches	(1) Study question	The prevalence of frequent headache among adolescents increased with decreasing socioeconomic status
Incledon et al. (2016)	*N* = 4983, *M*_age_ = 10.3 (range: 10–11), Chronic pain	Longitudinal	(1) Maternal mental health; (2) Mother's chronic pain; (3) Hostile parenting style; (4) Adverse life events; (5) Sociodemographic factors	(1) Kessler Scale; (2) Study question; (3) Study questions; (4) Study questions; (5) Study questions	(1) Chronic pain	(1) Study questions—Parent reported	Family factors assessed in early childhood did not predict chronic pain problems in later childhood
Ioannis et al. (2013)	*N* = 106, *M*_age_ = 8.4 (range: 5–14), RAP	Case–control	(1) Social family problems; (2) Financial problems; (3) Family moves to other locations in the last 3 years; (4) Stressful life events; (5) History of sexual abuse	(1–5) Study survey—parents reported	(1) RAP	(1) Clinical evaluation	Low socioeconomic status, family movement and severe economic difficulties were more frequent in children with RAP
Işik et al. (2009)	*N* = 2669, *M*_age_ = 8.2 (range: 6–12), Headache	Cross‐sectional	(1) Family history for headache; (2) Socioeconomic status	(1–2) Structured questionnaire	(1) Migraine	(1) Structured questionnaire	Headache was more common among children with lower socioeconomic groups
Juang et al. (2004)	*N* = 4645, *M*_age_ = NR (range: 13–15), Headache	Case–control	(1) Childhood family environment; (2) ACEs	(1–2) The Global Family Environment Scale	(1) Chronic daily headache	(1) The two‐stage headache survey	Childhood adversities might be contributing to greater risk of developing chronic daily headaches in young adolescents
Kaczynski et al. (2009)	*N* = 266, *M*_age_ = 13.3 (range: 8–17), Chronic pain	Cross‐sectional	(1) Protective parenting	(1) The Adult Responses to Children's Symptoms	(1) Current pain intensity; (2) Lowest pain; (3) Highest pain; (4) Depression; (5) Anxiety; (6) Passive coping; (7) Functional disability	(1–3) Semi structured interview; (4) Children's Depression Inventory; (5) Revised Children's Manifest Anxiety Scale; (6) Pain Response Inventory; (7) Functional Disability Inventory	The association between protective parenting and functional disability was relatively stronger in boys than in girls. However, this difference was not statistically significant
Kaczynski et al. (2013)	*N* = 262, *M*_age_ = 14.7 (range: 11–17), Headache and Migraine	Cross‐sectional	(1) Protective parenting	(1) The Adult Responses to Children's Symptoms	(1) Headache frequency; (2) Headache duration; (3) Headache severity; (4) Anxiety; (5) Depression; (6) Passive coping; (7) School functioning	(1–3) Clinical evaluation; (4) Revised Children's Manifest Anxiety Scale; (5) Children's Depression Inventory; (6) Pain Response Inventory; (7) PedsQL School Functioning scale	Protective parenting was associated with school‐related disability in adolescents with tension type headache
Kaczynski et al. (2016)	*N* = 382, *M*_age_ = 13.9 (range: 5–17), Headache	Cross‐sectional	(1) Family functioning; (2) Protective parenting	(1) Family Relationship Index; (2) Adult Responses to Children's Symptoms	(1) Pain duration; (2) Pain intensity; (3) Depression; (4) Functional disability	(1) 0–10 Numerical Rating Scale; (2) Clinical evaluation; (3) The Children's Depression Inventory, Second Edition; (4) Functional Disability Inventory	Family functioning and protective parenting style were associated with depression, but not disability. There was an indirect pathway from family functioning to depression to disability
Kandemir et al. (2018)	*N* = 50 children, *M*_age_ = NR (range 8–18), Migraine	Case–control	(1) Problem solving; (2) Communication; (3) Roles; (4) Affective responsiveness; (5) Affective involvement; (6) Behaviour control; (7) General family functioning; (8) Parenting style inventory; (9) Family structure	(1–7) Family Assessment Device; (8) Parenting Style Inventory; (9)	(1) Migraine	(1) Clinical evaluation according to International Classification of Headache Disorders–3 beta criteria	Family functioning, and particularly affective responsiveness, was associated with migraine in children and adolescents
Kashikar‐Zuck et al. (2008)	*N* = 93, *M*_age_ = 14.85 (range: 11–18), Juvenile Fibromyalgia Syndrome	Case–control	(1) Maternal pain history; (2) Maternal distress; (3) Family environment; (4) Family functioning	(1) Parent Pain History Questionnaire; (2) Symptom Checklist‐90‐Revised; (3, 4) Family Environment Scale	(1) Depressive symptoms; (2) Behaviour problems; (3) Self‐esteem; (4) Impact of JFS on daily functioning	(1) Children's Depression Inventory; (2) Child Behaviour Checklist; (3) Self‐Perception Profile for Adolescents; (4) Modified Fibromyalgia Impact Scale for Children	Mothers of adolescents with Juvenile Fibromyalgia Syndrome (JFS) reported twice as many pain conditions and significantly greater depressive symptoms than mothers of comparison peers without JFS. The group with JFS also had poorer overall family functioning and more conflicted family relationships. In adolescents with JFS, maternal pain history was associated with significantly higher functional impairment
Kristjánsdóttir (1996)	*N* = 2173, *M*_age_ = NR, (range: 11–15), Back pain	Cross‐sectional	(1) Social class; (2) Residence	(1–2) The Health Behaviour of School‐aged Children Survey	(1) Presence of weekly backache	(1) The Health Behaviour of School‐aged Children Survey	Older adolescents from rural areas reported significantly more back pain than those living in the city
Kristjánsdóttir and Wahlberg (1993)	*N* = 2140, *M*_age_ = NR (range: 11–16), Headache	Cross‐sectional	(1) Social class; (2) Residence	(1–2) The Health Behaviour of School‐aged Children survey	(1) Presence of weekly headache	(1) The Health Behaviour of School‐aged Children survey	There was no significant association between social class and weekly headache
Kröner‐Herwig et al. (2008)	*N* = 4043, *M*_age_ = NR (range: 9–14), Headache	Cross‐sectional	(1) Parent household; (2) Financial burden; (3) Working status of parents; (4) Socioeconomic status; (5) Family climate; (6) Family quarrelling; (7) Parental solicitousness; (8) Parental headache	(1–8) study survey	(1) Headache frequency	(1) Study survey	Parental headache increased the relative risk of headache in children and adolescents. Living in a single‐parent household and the family being financially strained (parents' subjective evaluation) were found to be predictive of headache. Financial burden was a risk for weekly headache
Langer et al. (2011)	*N* = 132, *M*_age_ = 11.36 (range:7–17), Abdominal pain	Cross‐sectional	(1) Protectiveness response	(1) Adults' Responses to Children's Symptoms	(1) Disability	(1) Functional disability inventory	Parent protectiveness predicted child functional disability
Langer et al. (2014)	*N* = 184, *M*_age_ = 13.72 (range: 8–18), Chronic pain	Cross‐sectional	(1) Parent catastrophizing; (2) Parental protective responses	(1) The Pain Catastrophizing Scale‐Parent; (2) Adult Responses to Children's Symptoms	(1) Child pain intensity	(1) Faces Pain Scale‐Revised	Parental catastrophizing and protective responses were not associated with child pain intensity
Larsson and Sund (2007)	N T1 = 2465, *M*_age_ = 13.7 (range: 13–17), Chronic pain	Longitudinal	(1) Parent socio‐economic status; (2) Parental divorce	(1–2) Study survey	(1) Regular and troublesome pain complaints (pain at least weekly)	(1) Study survey	Frequent back pain among adolescents was associated with parental divorce occurring during the last year
Laurell et al. (2005)	*N* = 130, *M*_age_ = 12.1 (range: 7–17), Headache	Case–control	(1) Family history of pain or other diseases; (2) Family history of migraine; (3) Parents' divorce; (4) Family structure; (5) Lack of money	(1–5) Survey questions	(1) Children headache	(1) Survey questions	Migraine among first‐degree relatives and the total sum of physical symptoms in children were the strongest predictors of headache in logistic regression analysis
Law et al. (2020)	*N* = 239, *M*_age_ = 14 (range: 11–17), Headache	Longitudinal	(1) Protective parenting behaviours at baseline; (2) Parental catastrophizing at baseline; (3) Parent headache‐related disability at baseline; (4) Parent headache frequency at baseline	(1) Protect subscale from the Adult Responses to Children's Symptoms questionnaire; (2) Pain Catastrophizing Scale‐Parent; (3) Headache Impact Test‐6; (4) Retrospective measure	(1) Adolescent headache‐related disability at follow‐up; (2) Adolescent headache frequency at follow‐up	(1) Paediatric Migraine Disability Assessment; (2) 28‐day online headache diary	Greater parental catastrophizing at baseline was a significant predictor of greater adolescent headache‐related disability at follow‐up. More frequent protective parenting behaviour and greater parental catastrophizing at baseline were significant predictors of increased adolescent headache frequency over time
Lewandowski and Palermo (2009)	*N* = 30, *M*_age_ = 13.5 (range: 11–16), Headache	Cross‐sectional	(1) Race/ethnicity; (2) Family income; (3) Parent education background; (4) Parent marital and work status; (5) Parent‐adolescent conflict; (6) Family functioning	(1–4) study questionnaire; (5) The Issues Checklist; (6) The McMaster Family Assessment Device	(1) Depressive symptoms	(1) Revised Child Anxiety and Depression Scale	Parent‐adolescent conflict and family functioning predicted depressive symptoms
Liakopoulou‐Kairis et al. (2002)	*N* = 129, *M*_age_ = 10.0 (range: 8–13), Chronic pain	Case–control	(1) Maternal psychopathology; (2) Parental expressed emotion; (3) Family functioning; (4) Negative life events	(1) The Symptom Checklist‐90‐R; (2) Parental Expressed Emotion; (3) The McMaster Family Assessment Device; (4) The Life Events Scale for Children	(1) RAP; (2) Recurrent headache	(1–2) Clinical evaluation	Mothers of children with RAP showed more symptoms for anger and hostility than chronic pain‐free controls. Mothers with children either with RAP or recurrent headache had higher expressed emotion than mothers of control children. Youth either with RAP or recurrent headache reported more negative life events than controls
Lindley et al. (2005)	*N* = 23, *M*_age_ = 14 (range: 8–15), Abdominal pain	Cross‐sectional	(1) Parental aggression/hostility; (2) Parental separation or divorce	Not reported	(1) Persistence of abdominal pain	(1) Clinical assessment	Parental aggression/hostility and parental separation or divorce were not associated with persistence of abdominal pain
Logan and Scharff (2005)	*N* = 78, *M*_age_ = 12.1 (range: 7–17), Chronic pain	Cross‐sectional	(1) Family size; (2) Maternal years of education; (3) Parental years of education; (4) Parental psychological distress; (5); Family environment	(1–4) Study questionnaire; (4) Revised Symptom Checklist‐90; (5) Family Environment Scale	(1) Pain intensity; (2) Pain‐free days; (3) Pain duration; (4) Functional disability	(1–2) Pain index; (3) Clinical evaluation; (4) Functional Disability Inventory	Parent distress and family characteristics were associated with functional disability
Lynch‐Jordan et al. (2018)	*N* = 303, *M*_age_ = 15.1 (range: 12–18), Chronic pain	Cross‐sectional	(1) Parent protective responses to child pain; (2) Parent monitoring responses to child pain; (3) Parent solicitousness responses to child pain; (4) Parent pain catastrophizing	(1–3) Adult Response to Children's Symptoms; (4) Pain Catastrophizing Scale (Parent Report)	(1) Pain intensity; (2) Functional disability; (3) Adolescent pain behaviours	(1) Visual analogue scale; (2) Functional Disability Inventory; (3) Adolescent Pain Behaviour Questionnaire	Greater pain behaviour was associated with increased protective responses, and greater protective behaviour was associated with increased disability
Lynch‐Jordan et al. (2013)	*N* = 240, *M*_age_ = 14.76 (range: 12–18), Chronic pain	Cross‐sectional	(1) Parent pain catastrophizing	(1) Pain Catastrophizing Scale for Parents	(1) Pain intensity; (2) Pain‐related disability; (3) Health‐related quality of life; (4) Depression symptoms; (5) Pain behaviours	(1) 0–10 Numeric rating scale; (2) Functional Disability Inventory; (3) Paediatric Quality of Life; (4) Children's Depression Inventory; (5) Adolescent Pain Behaviour Questionnaire parent reported	High catastrophizing parents had adolescents with significantly more depressive symptoms, greater functional disability, higher pain intensity, and greater pain behaviours than low catastrophizing parents
Mansuri et al. (2020)	*N* = 58,958, *M*_age_ = 10 (range: 3–17), Headache	Cross‐sectional	(1) Race; (2) Ethnicity; (3) ACEs	(1–3) Study survey	(1) History of frequent or severe headaches	(1) Clinical evaluation‐parent reported	Experiencing one or more ACEs compared to none was associated with a higher risk of headaches in children, and the difficulty due to family's income was the only ACE independently associated with recurrent/frequent headaches. No interaction between sex and ACEs was found
Marmorstein et al. (2009)	*N* = 674, *M*_age_ = 17.5 (range: 17–19), Headache	Cross‐sectional	(1) Parental major depressive disorder; (2) Parental antisocial behaviour; (3) Parental alcohol dependence; (4) Parental drug dependence	(1) Structured Clinical Interview for DSM‐III‐R; (2) Structured Clinical Interview for DSM‐III‐R Personality Disorders; (3–4) The Substance Abuse Module of the Composite International Diagnostic Interview	(1) Migraine headaches; (2) Stomach‐related problems	(1–2) Study question	Parental depression, antisocial behaviour and drug dependence were associated with offspring migraine. There were no significant associations between parental psychopathology and offspring stomach problems
Miró et al. (2019)	*N* = 111, *M*_age_ = 14.41 (range: 8–21), Chronic pain	Cross‐sectional	(1) Participant's perceived social support from family; (2) Perceived solicitous response from participant's parents	(1) Multidimensional Scale of Perceived Social Support; (2) West Haven‐Yale Multidimensional Pain Inventory Solicitous scale	(1) Pain interference; (2) Pain catastrophizing	(1) Brief Pain Inventory; (2) Pain Catastrophizing Scale	Children's perceptions of pain‐related solicitous responses from their parent/guardian were associated both with more pain interference and greater pain‐related catastrophizing. Perceived social support was negatively associated with pain interference
Moore et al. (2019)	*N* = 50, *M*_age_ = 6 (range: NR), Chronic pain	Cross‐sectional	(1) Mothers' number of pain locations; (2) Mothers' pain interference; (3) Maternal depressive symptoms at child aged 12‐months; (4) Maternal hope at child aged 12‐months	(1) Adapted structured pain questionnaire (2) Study question; (3) 20‐item Center for Epidemiologic Studies Depression scale; (4) Herth Hope Scale	(1) Offspring number of pain locations; (2) Offspring pain interference	(1) Adapted structured pain questionnaire; (2) Study question	Maternal hope but not depressive symptoms predicted fewer painful body regions in children five years later
Nelson et al. (2018)	*N* = 141, *M*_age_ = NR (range: 9–19), Chronic pain	Cross‐sectional	(1) Sociodemographic characteristics (age, gender, race/ethnicity, socioeconomic status, marital status); (2) Medical history	(1–2) Study questionnaire	(1) Chronic pain diagnosis; (2) ACEs; (3) Psychosocial functioning; (4) Pain‐related fear and catastrophizing	(1) Study questionnaire; 0–10 Numeric rating scale; FDI; (2) semi‐structured interview; (3) Children Depression Inventory 2, Revised Children's Manifest Anxiety Scale; (4) Fear of Pain Questionnaire, Pain Catastrophizing Scale	78% of youth with chronic pain reported at least 1 ACE in their lifetime. ACEs exposure was significantly associated with greater symptoms of anxiety, depression, and fear of pain, with ≥ 3 ACEs associated with greatest impairment in functioning. No association was found between ACEs and increased pain‐related disability or pain intensity
Nelson et al. (2021)	*N* = 155, *M*_age_ = NR (range: 10–18), Chronic pain	Longitudinal	(1) Sociodemographic characteristics (age, gender, race/ethnicity)	(1) Study questionnaire	(1) Pain; (2) ACEs; (3) Anxiety and Depression; (4) Quality of life	(1) Pain Questionnaire, 0–10 Numerical rating Scale; (2) CYW Adverse Childhood Experiences Questionnaire; (3) Revised Child Anxiety and Depression Scale (4) Paediatric Quality of Life Inventory	Youth with chronic pain with a history of 3+ ACEs reported significantly higher PTSD, depressive and anxiety symptoms, and poorer quality of life than youth with no ACE history. PTSD was found to moderate the association between ACEs and anxiety and depressive symptoms
Neufeld et al. (2013)	*N* = 1360, *M*_age_ = 8.1 (range: 1–16), JIA	Case–control	(1) Parental separation; (2) Death of a parent; (3) Illness of a family member; (4) Experience with a serious upset or serious loss at about the time of symptom onset; (5) Recent death of anyone close to the child; (6) Problems getting along with others; (7) Mother employed outside home; (8) Ethnic groups; (9) Urban/rural residence; (10) Living in a city	(1–10) Study questionnaire	(1) Presence of JIA	(1) Physician evaluation	There were significant associations between stressful life events like a currently ill family member, separated parents or difficulties with interpersonal relationships and occurrence of JIA
Oswari et al. (2019)	*N* = 1813, *M*_age_ = 13.54 (range: 10–17), Abdominal pain	Cross‐sectional	(1) Family size; (2) Birth order; (2) Mother employed; (3) Father's employment; (4) Stressful life events	(1–4) Study questionnaire	(1) Presence of functional abdominal pain disorders	(1) Questionnaire on Paediatric Gastrointestinal Symptoms	Family‐related stressful life events were associated with abdominal pain disorders in adolescents from Indonesia
Petri et al. (2024)	*N* = 1850, *M*_age_ = 7 (range: 3–13), Recurrent pain	Longitudinal	(1) Depression; (2) Pain; (3) SES	(1, 2) Patient Health Questionnaire; (3) Study questionnaire	(1) Pain; (2) Behavioural strengths and difficulties; (3) Puberty stages	(1) Gieben Complaints Form; (2) Strengths and Difficulties Questionnaire; (3) Tanner's Sexual Maturity Rating	Maternal pain, maternal depressiveness, and lower family socioeconomic status as well as child's emotional difficulties are significantly associated with a higher frequency of recurrent pain in children as perceived by their parents
Reid et al. (1997)	*N* = 45, *M*_age_ = 14.5 (range: 8–18), JRA and Fibromyalgia	Case–control	(1) Depression; (2) Anxiety; (3) Pain coping; (4) Family functioning; (5) Parents response to child pain	(1) Beck Depression Inventory; (2) State–Trait Anxiety Inventory; (3) Pain Coping Questionnaire; (4) Family Adaptability and Cohesion Evaluation Scales; (5) Illness Behaviour Encouragement Scale	(1) Functional disability; (2) School attendance; (3) Depression; (4) Anxiety; (5) Pain coping; (6) Family functioning; (7) Perceived parent pain responses; (8) Pain intensity; (9) Fatigue	(1) Functional Disability Inventory; (2) School report; (3) Children's Depression Inventory, Beck Depression Inventory; (4) State–Trait Anxiety Inventory for Children; (5) Pain Coping Questionnaire; (6) Family Adaptability and Cohesion Evaluation Scales; (7) Illness Behaviour Encouragement Scale; (8–9) 0–10 Numerical rating scale	Fibromyalgia is associated with disability of a magnitude comparable to that of other chronic pain conditions like JRA. Disability among children with fibromyalgia or JRA is a function of the children's psychological adjustment and physical state, and of the parents' physical state and method of coping with pain
Robins et al. (2005)	*N* = 62, *M*_age_ = 11.2 (range: 6–16), Abdominal pain	Cross‐sectional	(1) Parents psychological distress	(1) Symptom Checklist‐90‐R	(1) Dysfunctional dyspepsia; (2) Irritable bowel syndrome; (3) Functional abdominal pain	(1–3) Clinical assessment	Parents of children with dysfunctional dyspepsia had significantly higher psychological distress than parents of children with irritable bowel syndrome and parents of children with functional abdominal pain
Roman‐Juan et al. (2023)	*N* = 469, *M*_age_ =14.28 (range:13–18), Chronic pain	Cross‐sectional	(1) Adverse childhood events (ACEs)	(1) Center for Youth Wellness Adverse Childhood Experiences Questionnaire Teen Self‐Report (CYW ACE‐Q Teen SR	(1) Demographic variables (age, sex); (2) Chronic pain characteristics; (3) ACEs; (4) Sleep disturbance; (5) Anxiety and depression symptoms	(1) Study questionnaire; (2) Graded Chronic Pain Scale‐Revised; (3) CYW ACE‐Q Teen SR; (4–5) PROMIS	Sleep disturbance explained the association between exposure to ACEs and the presence of chronic pain. It also explained the variance in the association between exposure to ACEs and depressive symptoms and moderated the association between exposure to ACEs and anxiety in adolescents with chronic pain
Roman‐Juan et al. (2024)	*N* = 1115, *M*_age_ = 11.67 (range: 10–18), Chronic pain	Cross‐sectional	(1) Immigration background	(1) Study questionnaire	1) Demographic variables (age, sex, birth country and birth country of their parents); (2) Chronic pain intensity and frequency; (3) Pain‐related interference	(1) Study questionnaire; (2) Study questionnaire (pain checklist); (3) PROMIS	Having an immigration background was associated with a greater prevalence of chronic pain and high‐impact chronic pain; higher in children and younger adolescents than in older adolescents
Schanberg et al. (2021)	*N* = 89, *M*_age_ = 12.8 (range: 6–19), Rheumatic disease	Cross‐sectional	(1) Parental pain history; (2) Family pain history; (3) Parental pain intensity; (4) Parental disability; (5) Parental pain severity	(1–2) Pain history questionnaire; (3) Chronic Pain Grading Scale	(1) Current pain rating; (2) Global assessment; (3) Number of pain locations; (4) Catastrophizing	(1) Visual analogue scale; (2) Physician global assessment and self‐report questionnaires; (3) Body map; (4) Coping Strategies Questionnaire for Children	Parents reporting higher levels of current pain and higher mean levels of pain during the past month were more likely to have children reporting higher levels of current pain. Children whose families reported a history of multiple pain conditions were more likely to report higher levels of current pain intensity and more pain locations
Shaygan and Karami (2020)	*N* = 734, *M*_age_ = 14.95 (range: 12–19), Chronic pain	Cross‐sectional	(1) Mothers' education; (2) Fathers' education; (3) Mothers' job; (4) Fathers' job; (5) Parenting style	(1–4) Study questionnaire; (5) Baumrind parenting style questionnaire	(1) Presence of chronic pain	(1) Study questionnaire	Authoritarian parenting style and authoritative parenting style were significantly correlated with chronic pain after controlling for demographic variables
Shenoi et al. (2016)	*N* = 363, *M*_age_ = 11.1 (range: 8–15), JIA	Case–control	(1) Race; (2) Ethnicity; (3) Annual household income; (4) Perinatal characteristics; (5) Body mass index; (6) Cigarette smoke exposure; (7) Breastfed; (8) Duration breastfed; (9) Cow's milk introduction; (10) Hospitalisation for infection at age < 1 year; (11) Daycare attendance at age < 6 years; (12) Birth order; (13) Household pets; (14) Stressors; (15) Other risk estimates	(1–15) Study structured questionnaire	(1) Childhood JIA	(1) Clinical diagnosis	Compared to playmate‐matched controls, preterm delivery was associated with JIA. There was no association between JIA and household smoking or maternal prenatal smoking, breastfeeding, hospitalisation with infection in the first year of life, day‐care attendance before 6 years of age, household pets, or residential area prior to the onset of JIA
Sieberg et al. (2011)	*N* = 157, *M*_age_ = 13.7 (range: 8–17), Chronic pain	Cross‐sectional	(1) Parent somatization; (2) Parent depression; (3) Parent anxiety; (4) Parent depression in the context of children's pain; (5) Parent anxiety in the context of children's pain; (6) Parent catastrophizing in the context of children's pain; (7) Parent helplessness in the context of children's pain; (8) Parent protectiveness	(1–4) Brief Symptom Inventory‐18; (5–7) The Bath Adolescent Pain‐Parent Impact Questionnaire; (8) The Adult Responses to Children's Symptoms	(1) Child average pain intensity; (2) Child functional disability	(1) Semi‐structured interview with the clinical psychologist; (2) The Functional Disability Inventory	Protective parental responses partially mediated the relation between parent's distress and child functional disability for depression, anxiety, and catastrophizing. However, parental protective behaviour fully mediated the association with parent's helplessness and child's functional disability, indicating that feelings of parent helplessness did not uniquely contribute to the child's functional disability
Simons et al. (2008)	*N* = 217, *M*_age_ = 14.8 (range: 12–17), Chronic pain	Cross‐sectional	(1) Family socioeconomic status; (2) Parent responses to child pain behaviours	(1) Four‐factor index of social status; (2) Adult Responses to Adolescent's Symptoms	(1) Current pain intensity; (2) Coping; (3) Functional disability; (4) Somatization	(1) Semi‐structured interview with the clinical psychologist; (2) Pain Response Inventory; (3) Functional Disability Inventory; (4) Adolescent's Somatization Inventory	Passive and active coping interacted with parental protective behaviour to predict adolescents' pain behaviours. Among adolescents who reported infrequent use of passive or active coping strategies, higher levels of parental protective behaviour were associated with higher levels of disability and somatic symptoms
Siniatchkin et al. (2003)	*N* = 60, *M*_age_ = 10.5 (range: NR), Migraine	Case–control	(1) Parent control over problem‐solving behaviour in a stressful situation	(1) Behavioural marital therapy coding system	(1) Presence of migraine	(1) Clinical diagnosis	Communication with the affected child in migraine families was significantly more directive, with more specific instructions and less help, towards migraineurs than towards healthy siblings
Siu et al. (2012)	*N* = 1518, *M*_age_ = NR (range: 11–19), Chronic pain	Cross‐sectional	(1) Monthly income; (2) Father's education level; (3) Mother's education level; (4) Father's employment status; (5) Mother's employment status; (6) Parents' marital status; (7) Living condition; (8) Perceived social support, child reported	(1–7) Study questionnaire; The Multidimensional Scale of Perceived Social Support	(1) Chronic pain; (2) Sleep disturbances	(1) Study survey/Chronic Pain Grades; (2) The Pittsburgh Sleep Quality Index	Monthly income, having a parent with a part‐time employment, and lower perceived social support were associated with comorbid chronic pain and sleep disturbance
Solé et al. (2024)	*N* = 1121, *M*_age_ = 11.6, (range: 8–18), Chronic pain	Cross‐sectional	—	—	(1) Demographics (gender, age, and school level); (2) Pain location, intensity and frequency; (3) Pain interference; (4) Depressive symptoms and anxiety; (5) Bullying	(1) Study questionnaire; (2) Study questionnaire and 0–10 Numerical Rating Scale; (3–4) PROMIS; (5) Study questionnaire	There was a significant positive association between having a history of being a victim of bullying and having chronic pain
Stensland et al. (2014)	*N* = 7154, *M*_age_ = 16.0 (range: 12–18), Chronic pain	Cross‐sectional	(1) Interpersonal violence; (2) Family cohesion	(1) Young‐HUNT3 lifetime trauma screen; (2) Resilience Scale for Adolescents	(1) Loneliness; (2) Psychological distress; (3) Recurrent headache	(1) Study question; (2); The Symptom Checklist‐5; (3) Study question	Exposure to interpersonal violence was significantly associated with recurrent headache
Stone et al. (2018)	*N* = 154, *M*_age_ = 14.14 (range: 11–17), Abdominal pain	Cross‐sectional	(1) Parent chronic pain; (2) Parent self‐reported solicitousness	(1) Persistent Pain Questionnaire; Adult Responses to Children's Symptoms	(1) Pain threat appraisal; (2) Daily pain severity; (3) Daily functional impairment	(1) The Pain Beliefs Questionnaire‐Short Form; (2) 0–10 Numerical rating scale; (3) Child Activity Limitations Interview	Parental pain behaviours (i.e., modelling) had the strongest association with adolescents' subsequent daily pain severity and functional impairment in the context of parental chronic pain. Adolescents were aware of parent's pain behaviours, as indicated by the correspondence in path analysis outcomes between parent's self‐report or adolescent proxy‐report of parental pain behaviours
Tavasoli et al. (2013)	*N* = 380, *M*_age_ = NR (range: 4–15), Headache	Cross‐sectional	(1) Child abuse; (2) Family history of headache; (3) Low socioeconomic status	(1–3) Study questionnaire	(1) Presence of migraine headache; (2) Presence of tension‐type headache	(1–2) Clinical assessment	Child abuse was not associated with migraine. Children with migraine had higher odds ratio of family history of headache
Timko et al. (1993)	*N* = 204, *M*_age_ = 9.3 (range: NR), Rheumatic disease	Longitudinal	(1) Depression mood; (2) Drinking problems; (3) Parent's strain resulting from demands of the child's illness; (4) Social activities with friends; (4) Number of close relationships; (5) Parent's mastery	(1–5) Health and Daily Living Form; (3) Family Effects of Illness scale	(1) Pain intensity; (2) Functional disability; (3) Behaviour problems	(1) Visual analogue scale; (2) Child Health Assessment Questionnaire; (3) Child Behaviour Checklist	Parental risk and resistance factors at baseline predicted children's adjustment after age and baseline functioning were controlled. Mothers' and fathers' personal strain and depressed mood, and fathers' drinking problems, were associated with poorer children's adjustment; mothers' and fathers' social functioning appeared to aid patients' adjustment. Fathers' risk and resistance factors contributed independently from those of mother, to predict children's poorer functioning
Timmers et al. (2019)	*N* = 578, *M*_age_ = 15.2 (range: 12–18), Chronic pain	Cross‐sectional	(1) Parent psychological flexibility; (2) Parent response to children's pain behaviours	(1) Parent Psychological Flexibility Questionnaire; (2) Adult Responses to Children's Symptoms	(1) Pain intensity; (2) Pain location; (3) Functional disability; (4) School functioning; (5) Pain acceptance; (6) Pain catastrophizing; (7) Peer relationships; (8) Pain interference; (9) Anxiety; and (9) Depression	(1) Visual analogue scale; (2) Body area checklist; (3) Functional Disability Inventory); (4) Paediatric Quality of Life Inventory; (5) Chronic Pain Acceptance Questionnaire‐Adolescents; (6) Pain‐Related Cognitions Questionnaire for Children and Adolescents; (7–9) PROMIS	Parental protective responses were a significant predictor of child disability. Parent psychological flexibility indirectly affected child functioning through its association with the behaviour of both parents (i.e., protectiveness) and child pain acceptance
Toupin April et al. (2012)	*N* = 180, *M*_age_ = 10.2 (range: 2–18), JIA	Longitudinal	(1) Economic hardship; (2) Psychological distress	(1) Economic Hardship Questionnaire; (2) Symptom Checklist‐90R	(1) Health related quality of life	(1) Juvenile Arthritis Quality of Life Questionnaire	Higher caregiver perceived economic hardship and psychological distress were associated with worse health‐related quality of life of children
Unalp et al. (2007)	*N* = 633, *M*_age_ = 15.94 (range: 14–18), Headache	Cross‐sectional	(1) Separated parents; (2) Number of siblings; (3) Mother's education; (4) Father's education; (5) Socioeconomic status; (6) Family history of headache; (7) Mother with headache; (8) Father with headache; (9) Siblings with headache; (10) Headache of mother's parents; (11) Headache of father's parents; (12) Family history of migraine	(1–11) Study questionnaire	(1) Presence of migraine; (2) Presence of tension‐type headache	(1–2) Study questionnaire	Children with migraine had more siblings than children with tension‐type headache. Fathers and mothers of children with migraine had more education than parents of children with tension‐type headache. Children with migraine had a longer history of headache and migraine than children with tension‐type headache
Verstappen et al. (2015)	*N* = 923, *M*_age_ = 6.7 (range: 1–16), JIA	Cross‐sectional	(1) Socioeconomic status (SES)	(1) Nationwide Index of Multiple Deprivation	(1) Number of limited joints; (2) Disease duration; (3) Functional disability; (4) Disease activity; (5) Arthritis interference	(1) Physician's global visual analogue scale; (2) Medical interview, medical records; (3) Childhood Health Assessment Questionnaire, visual analogue scale; (4) Juvenile Arthritis Disease Activity Score; (5) Illness Perception Questionnaire, Child Health Questionnaire Parent Form 50	JIA disease activity scores did not differ between the 3 SES groups (low, middle and high‐SES). Individuals in the low‐SES group reported higher disability scores than the high‐SES group. The low‐SES group had a worse perception about the consequences of the disease and the effects of treatment than the high‐SES group. Patients from a low‐SES background reported more problems with daily activities and had a lower perception of the consequences of the disease than patients from a high‐SES background
Voerman et al. (2015)	*N* = 15,220, *M*_age_ = NR (range: 11–18), Chronic pain	Cross‐sectional	(1) Regular arguments between parents; (2) Parental divorce; (3) Physical abuse by parents	(1–2) Study survey; (3) Rotterdam Youth Monitor	(1) Presence of chronic pain	(1) The Pain Barometer	Physical abuse, sexual abuse, and family conflict were more common in adolescents with chronic pain. Physical abuse by parents and parental divorce were not significantly associated with chronic pain
Vuorimaa et al. (2011)	*N* = 142 *M*_age_ = 11.92 (range: 8–15), JIA	Cross‐sectional	(1) Depression; (2) Parental depressive and anxiety symptoms; (3) Parental self‐efficacy; (4) Parental illness management	(1) Beck Depression Inventory–Second Edition; (2) Hospital Anxiety and Depression Scale; (3) Parent's Arthritis Self‐Efficacy; (4) Visual analogue scale	(1) Trait anxiety; (2) Depression and mood; (3) Functional disability; (4) Pain; (5) Somatic complaints; (6) Self‐efficacy; (7) Active joint count	(1) State–Trait Anxiety Inventory; (2) Finnish version the Child Depression Inventory; (3) Childhood Health Assessment Questionnaire; (4) Structured Pain Questionnaire; (5) Child Behaviour Checklist; (6) CASE Scale; (7) Paediatric rheumatologist assessment	The child's pain was significantly associated with parental depressive symptoms, somatic symptoms, social functioning, self‐efficacy and parental perception of the child's coping, and to the child's general well‐being. Parental and child depressive and anxiety symptoms were significantly associated
Wager et al. (2020)	*N* = 2280, *M*_age_ = 12.95 (range: 10–19), Chronic pain	Cross‐sectional	(1) Socioeconomic status; (2) Immigration background	(1–2) Study questionnaire	(1) Presence of chronic pain	(1) Study questionnaire	Socioeconomic and migration status were not significantly associated with chronic pain
Welkom et al. (2013)	*N* = 127, *M*_age_ = 15.58 (range: 11–18), Chronic musculoskeletal pain	Longitudinal	(1) Parent protective responses	(1) Adult Responses to Children's Symptoms	(1) Pain catastrophizing; (2) Pain‐related disability	(1) Pain Catastrophizing Scale for Children; (2) Functional Disability Inventory	Parental protectiveness was indirectly associated with disability through pain catastrophizing at the initial visit and follow‐up. Decreases in parent protectiveness, potentially initiated through the initial evaluation were associated with lower levels of disability at follow‐up through pain catastrophizing
Williams et al. (2017)	*N* = 33, *M*_age_ = 11.24 (range: 8–15), Abdominal pain	Cross‐sectional	(1) Parent symptom talk; (2) Parent non‐symptom talk	(1–2) 5 min audio, transcribed and coded, of interaction between parents and their children	(1) Abdominal pain frequency, duration, and intensity; (2) Pain beliefs; (3) Child pain catastrophizing	(1) Abdominal Pain Index (2) The Security scale, Pain Beliefs Questionnaire; (3) Pain Response Inventory	Parent symptom‐related talk was associated with more child symptom complaints and parent non‐symptom‐related talk with fewer child complaints. The association between symptom talks and complaints was greater for children with high catastrophizing. Non‐symptom talks were associated with fewer complaints for children with high threat appraisals
Wilson et al. (2020)	*N* = 272, *M*_age_ = 13.47 (range: 11–15), Chronic pain	Case–control	(1) Presence of chronic pain	(1) Clinical assessment	(1) Pain frequency; (2) Pain intensity	(1) Pain Questionnaire	Adolescents reported increased pain frequency, pain intensity, and somatic symptoms from baseline to one year follow‐up in the group of parents with chronic pain reporting the highest levels of symptoms at both time points
Yadav and Yadav (2013)	*N* = 64, *M*_age_ = 12.75 (range: 8–18), JIA	Case–control	(1) Parent–child relationship	(1) Parent Child Relationship Scale	(1) JIA; (2) Number of active joints; (3) Disease duration; (4) Disease activity	(1–3) Clinical assessment; (4) C‐reactive protein	Both parents of children with JIA were significantly more demanding and mothers were overprotective and pampering with object rewards. There was a positive association between disease duration and parent's tendency to neglect their children and between persistence of disease activity with father's punishment behaviour or remaining indifferent

Abbreviations: JIA, Juvenile Idiopathic Arthritis; JRA, juvenile rheumatoid arthritis; *M*_age_, mean age; NR, not reported; PTSD, post‐traumatic stress disorder; RAP, recurrent abdominal pain.

### 3.2 Assessment of Bias and Quality of Evidence

The agreement between the three reviewers on scores of methodological quality was 98%. Any disagreement was mainly due to differences in interpretation of criteria, but consensus was reached without having to consult the fourth researcher.

Most of the studies in this review revealed a moderate or high risk of bias, and only 16 (13%) were found to be of good quality. The main shortcomings were found to be (1) the assessment of family‐related factors (e.g., these were often assessed with only a few questions, instead of using a comprehensive and validated questionnaire; *n* = 40, 31%) and (2) the follow‐up (e.g., the variables under study were not assessed more than once over time after baseline; *n* = 99, 80%).

### 3.3 Basic Information and Study Characteristics

There was considerable variability in the design of the articles identified: 16 were longitudinal studies (14%), 20 were case–control studies (18%), and 82 were cross‐sectional studies (68%). The studies reviewed included a variety of chronic pain conditions. However, the majority focused on headache (*N* = 36; 30%) and juvenile idiopathic arthritis (*N* = 20; 17%). The number of participants ranged from nine (Connelly et al. 2010) to 48,567 (Groenwald et al. 2020), with some samples including children as young as 1 year old (Aromaa et al. 1998; Crushell et al. 2003; Helgeland et al. 2010; Verstappen et al. 2015) and as old as 19 years old (Evans, Meldrum, et al. 2010; Marmorstein et al. 2009; Nelson et al. 2018; Schanberg et al. 2021; Shaygan and Karami 2020; Siu et al. 2012; Wager et al. 2020).

The studies in this review describe 26 family‐related factors, which include emotional, cognitive, behavioural, economic, and sociocultural factors. To summarise the findings, all these family‐related factors were categorised based on an extensive review of the literature and grouped and subsumed into three broad categories or units of analysis: (1) parental individual variables (these are variables that are related to one or both parents; e.g., parent anxiety and depressive symptoms); (2) dyadic variables (these are variables that link two actors, usually the father or the mother and the child; e.g., parenting style); and (3) context‐related variables (these are variables that are related to family and contextual characteristics; e.g., family functioning or socio‐economic factors). We used these three categories because they provide a comprehensive framework for understanding how family‐related factors influence chronic pain in children and adolescents. Moreover, they reflect how applied social psychology (Hawkinshire 1994) has classified the factors that can influence an individual's behaviour and actions within social interactions, including those with chronic pain (Miró 2003). Table 5 lists the 26 family‐related factors.

**TABLE 5:** Overview of the 2*6* family‐related factors.

**Parental individual variables** *Parental psychological functioning* Parental distress Parental mental health Parental expressed emotions *Parental cognitive characteristics* Parental catastrophic thinking Parents' acceptance of pain Parental psychological flexibility Parental education Parental personality Parental psychiatric disorders
**Dyadic variables** Parental responses to the chronic pain behaviours of their children Parental communication style Parents perception of their children vulnerability Attachment and bonding
**Context related variables** Pain in the family *Family functioning* Family cohesion Affective responsiveness Family conflict Autonomy and independence Family and social support Family communication Adverse childhood experiences (ACEs) *Socio‐economic factors* Socio‐economic status Living in rural or urban areas Ethnicity Immigration Parental employment Family structure

### 3.4 Parental Individual Variables

We identified 54 articles (45%) that studied the influence of parental individual variables. Most of the articles focused on parental psychological functioning, and some of them examined parental cognitive, emotional, personality, or psychiatric characteristics, and parental education.

#### 3.4.1 Parental Psychological Functioning

We identified 25 articles (21%) that studied parental psychological functioning. Most of them focused on anxiety and depression, but other variables such as personality, perception of vulnerability, positive experiences of parents, and mental health were also studied.

The findings from the studies reviewed revealed that a significant number of parents of children and adolescents with chronic pain experience psychological distress, including depressive and anxiety symptoms (e.g., Conte et al. 2003; Kashikar‐Zuck et al. 2008; Petri et al. 2024; Robins et al. 2005). Moreover, the severity of the parent's depressive or anxiety symptoms has been found to be significantly associated with the child's pain (Vuorimaa et al. 2011) and function; more specifically, it is associated with the child's level of disability (Kaczynski et al. 2016), psychological distress (Chow et al. 2016; Logan and Scharff 2005), adjustment to chronic pain (Timko et al. 1993), and functioning over time (Chow et al. 2016), including family functioning (Kaczynski et al. 2016), and health care use (Connelly et al. 2012).

Although most studies have focused on factors that have a detrimental effect on children and adolescents with chronic pain, such as anxiety and depressive symptoms, research has also shown that parental experiences of positive events, such as good mental health or daily uplifts, can serve as protective factors for children and adolescents with chronic pain. For example, in a study of 51 children and adolescents with chronic arthritis, Anthony et al. (2011) reported that a greater number of uplifts in parents was associated with fewer depressive symptoms in their children. This, in turn, improved children's psychological adjustment to chronic pain and was also associated with less disease activity (Anthony et al. 2011).

Parental expressed emotions can have either a negative or a positive effect on their children's chronic pain. For example, one study showed that mothers of children with recurrent abdominal pain or recurrent headaches commonly experience more symptoms of anger and hostility than controls (Liakopoulou‐Kairis et al. 2002). Conversely, emotions such as hope can serve as emotional resilience factors. This was revealed by a study that found a significant positive association between parental hope and a reduction in painful body regions in their children with chronic pain (Moore et al. 2019).

#### 3.4.2 Parental Cognitive Characteristics

We identified 14 articles (12%) that examined the influence of parental cognitive characteristics on how children cope with and respond to chronic pain, and most of them (*N* = 13; 93%) focused on the role of parents' catastrophizing about their child's pain. Four of the articles found a strong significant association between parental pain catastrophizing and children and adolescents' pain‐related characteristics. For example, one study (Dougherty et al. 2021) reported a significant positive association between parental pain catastrophizing and the child's functional disability, maladaptive pain coping skills, and pain‐related stress. A further three articles found that higher levels of pain catastrophizing in parents regarding their children's pain were significantly associated with worse outcomes in their children with chronic pain. For example, children with parents that endorsed higher levels of pain catastrophizing reported more depressive symptoms and exhibited more pain‐related behaviours than those with parents that endorsed lower levels of pain catastrophizing (Dougherty et al. 2021; Feinstein et al. 2018; Lynch‐Jordan et al. 2013). In addition, children with parents endorsing higher levels of pain catastrophizing also reported higher pain intensity and pain interference levels (Dougherty et al. 2021) than those with parents endorsing lower levels of pain catastrophizing. However, research has also shown that higher levels of parental pain catastrophizing only partially account for the association between the child's pain intensity and pain catastrophizing (Cunningham et al. 2014). Some data showed a positive association between parental catastrophic thinking about pain and the way their children cope with pain. For example, Lynch‐Jordan et al. (2013) in a study of 303 adolescents with chronic pain found a concordance in the use of catastrophic thinking about pain in both parents and parent–child dyads, which suggests a tendency to use a similar style of coping with pain. In addition, research has shown that there is a significant sex‐related difference in the association between parental catastrophizing and their children's chronic pain. For example, two studies have shown that the mother's catastrophic thinking has a greater impact on their child's pain intensity and psychological function (Akbarzadeh et al. 2021; Hechler et al. 2011).

Moreover, parental catastrophic thinking, whether about their own pain or their children's, significantly predicts self‐reported protective responses to their children's pain (i.e., parents responding in ways that encourage sick behaviours) (Langer et al. 2011) and parental solicitous responses to their children's pain behaviours (i.e., positive reinforcement of these behaviours) (Hechler et al. 2011). These responses to children's pain and pain behaviours have been associated with a worse physical and psychological function in children with chronic pain (Chow et al. 2016; Hechler et al. 2011; Huguet and Miró 2008; Law et al. 2020; Lynch‐Jordan et al. 2018).

Parental cognitive characteristics can also be protective factors for chronic pain in their children and adolescents. We found evidence in three studies that reported significant positive associations between parents' acceptance of pain and their children's functioning, including less catastrophic thinking (Feinstein et al. 2018), lower daily activity avoidance (Beeckman, Simons, et al. 2019), and greater acceptance of pain by children (Timmers et al. 2019). Another parental cognitive factor that has been found to have a protective role is psychological flexibility. We identified two recent studies showing that parental psychological flexibility indirectly contributed to improved function, less disability, and lower levels of negative affect in children with chronic pain (Beeckman, Hughes, et al. 2019; Timmers et al. 2019).

#### 3.4.3 Parental Education

The experience of chronic pain in children and adolescents depends significantly on the educational background of their parents (Boey et al. 2000). We identified five articles (4%) that studied the influence of parental education on their children's pain. The findings show that poorer parental education is significantly and positively associated with a higher risk of children developing chronic pain, increased persistence of pain, and fewer economic and sociostructural resources such as access to healthcare or social support for solving pain problems (Aasland et al. 1997; Bener et al. 2000; Brady‐Fryer et al. 2004; Grasaas et al. 2023; Hoftun et al. 2013).

#### 3.4.4 Parental Personality and Psychiatric Disorders

In this section, we compile and report the results about how parental personality and psychiatric disorders are related to chronic pain in children and adolescents. We identified six related articles (5%).

It is worth noting that our search only found studies on the adverse effects of psychiatric disorders and parental personality traits on the chronic pain experience in children. Research has shown a higher prevalence of psychiatric disorders (e.g., hypochondriasis and schizophrenia) in the parents of children with conditions such as migraine or abdominal pain (Esposito et al. 2013; Feldman et al. 2010; Galli et al. 2009). In terms of parental personality traits, we found one study reporting that parental antisocial behaviour and drug dependence were associated with a higher prevalence of migraine in their offspring (Marmorstein et al. 2009) and another one showing that children with migraine without aura tended to have mothers who scored significantly higher on traits such as social introversion, obsessiveness, and cynicism (Esposito et al. 2013).

### 3.5 Dyadic Variables

As mentioned, dyadic variables link two actors, usually the parent(s) and the child. We identified 27 articles (23%) on dyadic variables. Of these, 19 studied parental attitudes or parental responses towards their children's chronic pain behaviours, four examined the association between attachment (i.e., the emotional bond with another person) and parental bonding (i.e., the attachment between the child and the parent), one investigated parental communication, and three explored parental perceptions of their child's vulnerability and self‐efficacy in coping with pain.

#### 3.5.1 Parental Responses to the Chronic Pain Behaviours of Their Children

The findings of the 19 studies reviewed (16%) show that parental protective responses (i.e., responses that either positively reinforce pain complaints through increased parental attention or presence or negatively reinforce pain complaints through permission to avoid unwanted roles or responsibilities), also known as parental solicitous responses (Huguet and Miró 2008), are associated with the chronification of their children's pain (Cordts et al. 2016; Ertem et al. 2019; Shaygan and Karami 2020) and with increased levels of pain intensity and pain‐related disability in children with chronic pain (Kaczynski et al. 2009, 2013; Langer et al. 2011, 2014; Lynch‐Jordan et al. 2018; Welkom et al. 2013). Furthermore, several studies show that these protective responses partially mediate the association between parental distress and their children's functional disability and mediate the association between parental helplessness and their children's functional disability (Cunningham et al. 2014; Sieberg et al. 2011). In addition, parental protective behaviour has emerged as a significant predictor of pain‐related fear, avoidance of activities, and deterioration of school functioning in children and adolescents with chronic pain (Chow et al. 2016). In relation to parental solicitous responses (i.e., responses offering assistance and taking over the child's chores), these have been shown to be associated with greater pain interference and pain‐related catastrophizing (Miró et al. 2019) as well as with higher levels of self‐reported disability in adolescents with chronic pain (Lynch‐Jordan et al. 2018).

Parental minimising responses (i.e., criticising the child's pain behaviours as excessive, also known as discouraging responses; Huguet and Miró 2008) have been found to be associated with increased somatic symptoms in children with chronic pain but not with functional disability (Hammond et al. 2019; Lynch‐Jordan et al. 2018; Simons et al. 2008).

Greater use of parental distracting and monitoring responses (i.e., the actions parents take to keep track of their child's pain) has been found to be associated with greater reductions in the child's activity and a lower positive mood level (Connelly et al. 2010; Cunningham et al. 2014).

Finally, a study conducted with a sample of children and adolescents experiencing migraine revealed noteworthy differences in parental communication. More specifically, parents adopted a more directive communication style when interacting with their children who had migraines. They gave them more specific instructions and less assistance than when interacting with siblings that did not have chronic pain (Siniatchkin et al. 2003). One study found differences between fathers' and mothers' responses to their children's pain behaviours, with fathers being more demanding and mothers overprotective (Yadav and Yadav 2013). It is interesting to note that the child's sex has been found to moderate the association between parental protective behaviours and the pain intensity of the child, with girls reporting higher levels of pain intensity in association with their parents enacting more protective behaviours (Clementi et al. 2019).

Although most studies have focused on parental responses that negatively impact on their children with chronic pain, research has also studied more appropriate parental responses to their children's pain that have a positive impact because they promote well‐behaviours and coping (Huguet and Miró 2008) such as parent pain acceptance or encouragement of non‐pain‐related behaviours. For example, in a study of children with chronic pain, Reid et al. (1997) found that participants with parents who encouraged them to engage in daily activities despite their pain had less functional disability.

#### 3.5.2 Attachment and Perception of Child Vulnerability

We identified five articles (4%) focusing on the influence of attachment (i.e., an emotional bond with another person) and parental bonding (i.e., an attachment between the child and the parent) in children and adolescents with chronic pain. This research has shown that attachment insecurity is associated with chronic pain in children and adolescents (Bizzi et al. 2020). Moreover, studies have reported strong negative associations between the attachment insecurity of parents and anxiety in their children with chronic pain (Bizzi et al. 2020; Williams et al. 2017). Unhealthy parental bonding, characterised by low levels of care and high levels of control from both mothers and fathers, has been found to be associated with increased pain and depression symptoms in adolescents with chronic pain (Evans et al. 2018).

Finally, we identified four articles studying caregivers' perception of their children's vulnerability and self‐efficacy to cope with pain. The data showed a positive association between parents' perception of child vulnerability and more health care utilisation (Connelly et al. 2010), increased child depressive symptoms and anxiety (Anthony et al. 2011), worse functional outcomes (Gaultney et al. 2017), and more protective parental responses (Dupen et al. 2016).

### 3.6 Context‐Related Variables

We identified 50 articles (42%) that studied how family and contextual factors were associated with function in children and adolescents with chronic pain. Most of these articles examined pain in the family, family functioning, and socio‐economic factors, but childhood experiences (ACEs) and school‐related factors were also studied.

#### 3.6.1 Pain in the Family

We identified 25 articles (21%) that studied the family history of pain, a key factor in understanding the intergenerational transmission of chronic pain. This section of the review examines how parental pain models contribute to the persistence and characteristics of chronic pain in children and adolescents.

The data showed a significant association between the parental model of chronic pain (i.e., the process by which pain behaviours are learned, with parents acting as models for their children's pain behaviours) and the persistence of chronic pain (Aasland et al. 1997; Bener et al. 2000; Graungaard et al. 2016; Işik et al. 2009), higher levels of pain intensity (Clementi et al. 2019; Schanberg et al. 2021), greater pain extent (i.e., number of pain locations, (Coenders et al. 2014; Hoftun et al. 2013) and higher pain frequency in children (Aromaa et al. 1998; Arruda et al. 2010; Bener et al. 2000; Laurell et al. 2005; Petri et al. 2024)).

Moreover, the findings from the studies reviewed show that children with parents who experience chronic pain have an increased risk of developing chronic pain themselves (Kröner‐Herwig et al. 2008; Tavasoli et al. 2013; Unalp et al. 2007; Stone et al. 2018). They are also more likely to experience other adverse outcomes, including symptoms of post‐traumatic stress disorder and a diminished health‐related quality of life (Beveridge et al. 2018; Stone et al. 2018; Wilson et al. 2020).

The data also showed that children tend to experience chronic pain at a younger age when there is a parental history of chronic pain (Eidlitz‐Markus et al. 2015) and that they generally develop their symptoms at a significantly younger age than their parents (Eidlitz‐Markus and Zeharia 2017). An earlier onset of pain supports the idea that family history, especially parental experiences with chronic pain, plays a key role in determining when pain begins in children and adolescents.

There is a sex‐related effect in the association between the parents' pain and their children's pain characteristics, with the data indicating that mothers are the most frequently affected parent in this context (Bener et al. 2000; Bode et al. 2003). Mothers of adolescents with chronic pain have been found to report twice as many chronic pain conditions (Incledon et al. 2016; Kashikar‐Zuck et al. 2008), greater functional impairment (Kashikar‐Zuck et al. 2008), and a greater likelihood of pain symptoms than mothers of children who have recovered from chronic pain (Crushell et al. 2003). Of particular note, the health status of the mother and her own chronic pain experience have also been associated with her evaluation of her child's pain. Mothers experiencing chronic pain are five times more likely to report that their child is in pain than mothers without chronic pain (Graungaard et al. 2016).

One study found significant sex differences in the way parental models of pain are associated with the development of chronic pain in adolescent boys and girls. In this study with 219 adolescents, the sex‐specific concordance rate between the incidence of chronic pain in mothers and daughters was found to be higher than in mothers and sons (Evans, Meldrum, et al. 2010). In addition, maternal reports of pain and psychological functioning were also significantly associated with their daughter's pain reports and psychological functioning but not with the pain reports of their sons. Moreover, daughters were more susceptible to developing pain in the same locations as their mothers. In contrast, a high percentage of boys' pain was related to their fathers' pain, suggesting that boys are more likely to mirror their fathers' pain than their mothers' (Evans, Meldrum, et al. 2010).

#### 3.6.2 Family Functioning

We identified 10 articles (8%) that focused on the influence of family functioning on the child's pain experience. The findings from these studies showed a strong association of several measures of family functioning with chronic pain in children and adolescents (Chaney and Peterson 1989; Feldman et al. 2010; Liakopoulou‐Kairis et al. 2002), but not with disease activity (Chaney and Peterson 1989). For example, family cohesion and affective responsiveness were found to be associated with better adjustment to chronic pain and reduced illness‐related concerns in children and adolescents, whereas family conflict was found to be associated with poorer adjustment to chronic pain and greater illness‐related concerns (Kandemir et al. 2018; Stensland et al. 2014). Moreover, higher levels of conflict, poorer overall family functioning, and lower levels of autonomy were associated with a higher prevalence of headache in adolescents and with more depressive symptoms in adolescents with headache (Bener et al. 2000; Kaczynski et al. 2016; Lewandowski and Palermo 2009). In adolescents, perceptions of family functioning and social support were found to be significant predictors of global self‐worth and depression (Cuneo and Schiaffino 2002), and family function was significantly associated with adolescent adjustment to pain and medication compliance (Chaney and Peterson 1989).

In addition, the data from these studies show that families of children with chronic pain were more dysfunctional in terms of communication among family members, affective responsiveness, family involvement, family organisation, role expectation, and overall family functioning (Conte et al. 2003; Liakopoulou‐Kairis et al. 2002).

It is important to note that family environment has been found to be associated with children's ability to function with pain. For example, lower levels of family independence and higher family conflict were associated with functional disability in children with chronic pain (Logan and Scharff 2005). Similarly, in a study with children and adolescents with juvenile primary fibromyalgia syndrome, participants reported higher levels of conflict in their family relationships (Kashikar‐Zuck et al. 2008). In addition, unhappiness in the family was found to be predictive of migraine episodes in children (Anttila et al. 1999).

The data also show that a positive family climate is a protective factor that reduces the risk of chronic pain in children and adolescents. For example, the family's ability to organise daily activities in a meaningful way for all members was found to be predictive of a high quality of life in adolescents with chronic pain (Frare et al. 2002; Kröner‐Herwig et al. 2008).

#### 3.6.3 Adverse Childhood Experiences (ACEs)

We identified 16 articles (13%) that studied adverse childhood experiences (i.e., the exposure to potentially traumatic experiences in childhood or adolescence, e.g., parental divorce or death of a close family member) in relation to chronic pain and function in children and adolescents. For example, parental divorce, financial hardship, living in an environment with alcohol or drug‐related issues, or with someone with mental illness, witnessing violence at home or in the neighbourhood, suffering physical neglect, living with someone who was suicidal, hospitalisation or death of a close family member, and parental incarceration have all been found to be associated with chronic pain in children and adolescents (Baiden et al. 2021; Boey and Goh 2001b; Feldman et al. 2010; Fuh et al. 2010; Groenwald et al. 2020; Larsson and Sund 2007; Liakopoulou‐Kairis et al. 2002; Lindley et al. 2005; Mansuri et al. 2020; Marmorstein et al. 2009; Nelson et al. 2018, 2021; Oswari et al. 2019; Roman‐Juan et al. 2024; Stensland et al. 2014; Voerman et al. 2015). In fact, the data show that youths with chronic or recurrent pain have experienced more ACEs than youths without chronic pain (Groenwald et al. 2020; Liakopoulou‐Kairis et al. 2002). For example, one study found that the prevalence of self‐reported sexual abuse in girls with recurrent headaches was twice that of their headache‐free peers (Stensland et al. 2014). Finally, ACEs in childhood have been found to be associated with chronic pain in early adolescence (Incledon et al. 2016; Juang et al. 2004).

#### 3.6.4 Socio‐Economic Factors

We found 39 articles (33%) about the relation between socio‐economic factors and chronic pain in children and adolescents. Most of the studies focused on socioeconomic status (SES) and residence location and showed that a low SES was more common in youths with chronic pain. This lower SES was also associated with a more negative perception of the consequences of chronic pain and with reporting more problems with daily activities (Barus et al. 2023; Abdul‐Sattar, Abou, et al. 2014; Anttila et al. 1999; Arruda et al. 2010; Arruda and Bigal 2012; Boey et al. 2000; Boey and Goh 2001a; Holstein et al. 2018; Ioannis et al. 2013; Işik et al. 2009; Kröner‐Herwig et al. 2008; Petri et al. 2024; Siu et al. 2012; Tavasoli et al. 2013; Toupin April et al. 2012; Verstappen et al. 2015). The findings show that high‐SES families have a more positive perception of the interference of chronic pain in daily activities and a more positive view of the effects of treatment than low‐SES families.

In addition, the findings of the studies identified show that children and adolescents with chronic pain living in rural areas report significantly higher rates of chronic pain and impaired health‐related quality of life than children and adolescents with chronic pain living in urban areas (Abdul‐Sattar, Elewa, et al. 2014; Boey et al. 2000; Graungaard et al. 2016; Kristjánsdóttir 1996; Kristjánsdóttir and Wahlberg 1993). Furthermore, children with chronic pain and a low SES or living in rural areas also reported lower school attendance (Abdul‐Sattar, Elewa, et al. 2014). One study found that living in an urban environment could be a positive socioeconomic factor for older adolescents with chronic pain, who reported significantly lower rates of chronic pain than their counterparts living in rural areas (Kristjánsdóttir 1996).

Other articles studied racialized groups, immigration, parental employment, and family structure. For example, four studies found that individuals of racialized groups were more likely to report chronic pain (Evans, Meldrum, et al. 2010; Ghandour et al. 2004; Neufeld et al. 2013). Moreover, children from racialized groups exhibited worse functioning than white children: for example, one study found that minoritized children, compared to white children, reported reduced sleep, lower global health, higher functional disability, increased somatisation, and pain intensity (Evans, Taub, et al. 2010). A recent study showed that the rates of chronic pain in children and young adolescents were higher in students with an immigration background than in those with a local background (Roman‐Juan et al. 2024). The family structure also has an important role in the chronic pain experienced by children and adolescents, and one study showed that adolescents living in a single‐parent or reconstructed family have higher rates of musculoskeletal pain than adolescents living in two‐parent families (Heikkala et al. 2023). Finally, parental unemployment, a mother employed outside the home, or a father with part‐time employment have been associated with higher rates of chronic pain in children and adolescents (Anttila et al. 1999; Bode et al. 2003; Evans, Meldrum, et al. 2010; Ioannis et al. 2013; Kröner‐Herwig et al. 2008; Neufeld et al. 2013; Siu et al. 2012).

## 4 Discussion

This systematic review synthesised the findings of 119 articles examining the association between chronic pain in children and adolescents and a wide variety of family‐related factors.

The methodological quality of the studies in the review was highly variable. Generally speaking, however, most of the studies were of moderate quality at best. For example, most studies included small samples (e.g., Gmuca et al. 2019), with participants from a wide age range (e.g., Baiden et al. 2021), and very few showed details for each age group (e.g., Baiden et al. 2021), thus making it difficult to associate the findings with specific ages. The fact that there are so many studies (88%) with methodological issues shows how difficult it is to draw general conclusions from the literature. Our analysis revealed variability in the evidence quality across family‐related factors. The main limitations were the following: (1) many studies used limited or unvalidated measures rather than comprehensive, validated questionnaires and (2) most studies lacked longitudinal follow‐up, measuring variables only once at baseline without tracking changes over time. These issues were particularly common in studies on parental individual factors, such as emotional and psychological variables, where evidence quality was often weaker. In contrast, studies assessing more objective factors, like socioeconomic status and family functioning, generally had stronger methodological quality. These findings highlight the need for more rigorous, comprehensive, and longitudinal research to better understand family‐related influences on chronic pain in children and adolescents. Furthermore, the predominance of cross‐sectional or retrospective designs limits the ability to draw causal inferences about pain development or maintenance. That said, however, this review shows significant associations between parental individual variables, dyadic variables, and context‐related variables, and some key pain‐related outcomes, including pain chronification, pain intensity, pain frequency, pain extent, pain‐related interference, and disability in children and adolescents.

The 119 articles examined 26 family‐related factors, including parental individual variables, dyadic variables, and context‐related variables. However, most studies (43%) focused on parental variables and particularly on depression and anxiety symptoms, pain in the family, and catastrophizing about their children's pain. The studies on these issues showed the lowest quality of bias in their associations with pain and function in children and adolescents with chronic pain.

Fifty‐four of the 119 studies show that parental individual factors play a critical role in the risk of worse pain outcomes and disability as well as in their ability to adapt to living with chronic pain. For example, parental individual factors have been associated with the chronification of children's pain (Cordts et al. 2016) and the development of pain‐related disability (Dougherty et al. 2021; Kaczynski et al. 2016). Conversely, specific parental individual factors can also serve as protective factors for children and adolescents with chronic pain (e.g., parent's psychological flexibility and resilience; Beeckman, Simons, et al. 2019).

The studies in this review show that there is also a significant association between dyadic variables, specifically parental responses towards their children's pain and the chronification of their children's pain (e.g., Shaygan and Karami 2020), pain intensity (e.g., Evans et al. 2018), and pain‐related disability (e.g., Miró et al. 2019). It is also important to acknowledge that while many of the studies identified emphasise the significant impact of parental factors on their children's pain, chronic pain in children and adolescents can also significantly affect and influence parents.

There are also significant associations between the context‐related variables and key pain‐outcome variables (e.g., family functioning, socioeconomic factors and ACEs; Clementi et al. 2019). Specifically, these context‐related variables are associated with the persistence of chronic pain (e.g., Graungaard et al. 2016), pain intensity (e.g., Schanberg et al. 2021), pain frequency (Aromaa et al. 1998), pain extent (e.g., Coenders et al. 2014), and functional impairment (Logan and Scharff 2005). On the other hand, good family cohesion and affective responsiveness are associated with better adjustment to chronic pain and fewer illness‐related concerns (Kandemir et al. 2018), and a higher SES can act as a protective factor against the negative effects of chronic pain (Verstappen et al. 2015).

These findings underscore the importance of contextual factors in understanding, assessing, and managing chronic pain in children and adolescents (Chambers 2003; Palermo and Chambers 2005). They support Palermo & Chambers's integrative model, which emphasises psychosocial aspects and the interplay of individual, dyadic, and context‐related influences on outcomes. Our review confirms that parental, dyadic, and contextual factors significantly impact children's pain, offering a nuanced synthesis of family‐related influences while highlighting the parent–child relationship's importance alongside individual factors. It also identifies methodological challenges, such as small sample sizes and the limited age‐specific data, which constrain definitive conclusions.

### 4.1 Recommendations for Future Research

The methodological quality of the studies reviewed was moderate at best. There is, therefore, a need for high quality studies that include a good selection (e.g., representativeness of the exposed cohort), comparability (e.g., using valid and reliable exposure measures), and exposure procedures (e.g., implementation of adequacy of follow‐up cohorts). Future studies must balance representation across children and adolescents' age groups, include diverse genders (boys, girls, and gender‐diverse individuals), and incorporate racialised groups. This approach should address structural inequities like systemic racism, socioeconomic disparities, and barriers to healthcare access (Hood et al. 2023) as well as the cultural and social experiences of families, including parent/family experiences of discrimination and the challenges they face in accessing care.

It is important to note that almost all the articles identified focused on the negative impact of family‐related factors. Thus, research studying modifiable protective factors is also needed to develop preventive and intervention programs for chronic pain in children and adolescents. Likewise, most of the research was cross‐sectional. Therefore, longitudinal studies are also required that have long‐term follow‐ups and validated instruments that assess whether family‐related factors can predict variation in chronic pain characteristics in children and adolescents. These studies would help to clarify the association between family‐related factors and the impact of chronic pain on children's lives over time. Most studies used small samples‐often fewer than 100 participants and sometimes as few as 30 participants. Future research should use larger samples with adequate representation of relevant subgroups to ensure sufficient statistical power. This would also allow for better examination of how family‐related factors influence age, gender, and socioeconomic status. Most of the studies reviewed used self‐report questionnaires to assess the variables of interest. Therefore, other approaches to assessment using other procedures, particularly observation, are also needed, as are studies that include ratings of both parents and children. This is important because the preliminary data show that it was the incongruence in the reports of family function between family members, and not the ratings per se, that predicted disability among the children participating in the study (Schanberg et al. 1998).

The bidirectional association between parental factors and their children's chronic pain warrants further research. Understanding how a child's pain affects parents' coping, emotions, and mental health is essential for developing holistic interventions addressing child and family needs. Future research should examine the impact on parents' well‐being and how these factors worsen pain outcomes. Studies (e.g., Liakopoulou‐Kairis et al. 2002) show that parental anger can intensify child pain, underlining a complex mutual influence warranting deeper exploration for comprehensive treatment strategies.

No studies addressed family members beyond parents (e.g., siblings and grandparents), necessitating further research on their impact on family dynamics and how children adjust to and cope with chronic pain. An emerging trend in the data underscores the significant role of parental individual, dyadic, and broader contextual factors in shaping pain outcomes in children and adolescents with chronic pain. This supports the biopsychosocial model of pain, suggesting that interventions should address not only the child's pain but also parental mental health, responses to the child's pain, and socio‐cultural influences. Some studies (e.g., Cordts et al. 2016) indicate that certain parental behaviours may act as protective factors, while others can have negative impacts, highlighting discrepancies that require further investigation to determine which behaviours are truly beneficial or harmful. Existing treatments, like cognitive behavioural therapy (CBT) for adolescents with migraine headaches (Power et al. 2013) and juvenile fibromyalgia (Kashikar‐Zuck et al. 2018, 2021), already incorporate parents, showing positive outcomes in managing both the child's pain and family dynamics. Nevertheless, there is a need for more comprehensive, family‐centered interventions that integrate a wide range of family‐related factors. Future strategies should consider T parental mental health, dyadic relationships, and socioeconomic context to develop personalised and effective treatment plans that improve both pain management and overall family coping strategies.

### 4.2 Limitations

This systematic review has several limitations. First, the inclusion criteria were broad and the studies included were highly heterogeneous. This, in addition to the very few studies on some variables, interfered with our ability to conduct a meta‐analysis. Second, despite a comprehensive and robust search strategy, some studies may have been missed. Third, limiting the search to English and Spanish publications might have excluded significant research in other languages.

Despite limitations, this review draws important conclusions about the role of family‐related factors in the development and maintenance of chronic pain and in the function of children and adolescents with chronic pain. These findings can guide the creation of more effective treatment and preventive programmes.
